# Combinations of Single Chain Variable Fragments From HIV Broadly Neutralizing Antibodies Demonstrate High Potency and Breadth

**DOI:** 10.3389/fimmu.2021.734110

**Published:** 2021-09-16

**Authors:** Rebecca T. van Dorsten, Kshitij Wagh, Penny L. Moore, Lynn Morris

**Affiliations:** ^1^Center for HIV and STIs, National Institute for Communicable Diseases of the National Health Laboratory Service, Johannesburg, South Africa; ^2^Medical Research Council (MRC) Antibody Immunity Research Unit, Faculty of Health Sciences, University of the Witwatersrand, Johannesburg, South Africa; ^3^Theoretical Division, Los Alamos National Laboratory, Los Alamos, NM, United States; ^4^Center for the AIDS Programme of Research in South Africa (CAPRISA), University of KwaZulu-Natal, Durban, South Africa

**Keywords:** HIV, broadly neutralizing antibodies, single chain variable fragments, combinations of scFv, HIV prevention

## Abstract

Broadly neutralizing antibodies (bNAbs) are currently being assessed in clinical trials for their ability to prevent HIV infection. Single chain variable fragments (scFv) of bNAbs have advantages over full antibodies as their smaller size permits improved diffusion into mucosal tissues and facilitates vector-driven gene expression. We have previously shown that scFv of bNAbs individually retain significant breadth and potency. Here we tested combinations of five scFv derived from bNAbs CAP256-VRC26.25 (V2-apex), PGT121 (N332-supersite), 3BNC117 (CD4bs), 8ANC195 (gp120-gp41 interface) and 10E8v4 (MPER). Either two or three scFv were combined in equimolar amounts and tested in the TZM-bl neutralization assay against a multiclade panel of 17 viruses. Experimental IC_50_ and IC_80_ data were compared to predicted neutralization titers based on single scFv titers using the Loewe additive and the Bliss-Hill model. Like full-sized antibodies, combinations of scFv showed significantly improved potency and breadth compared to single scFv. Combinations of two or three scFv generally followed an independent action model for breadth and potency with no significant synergy or antagonism observed overall although some exceptions were noted. The Loewe model underestimated potency for some dual and triple combinations while the Bliss-Hill model was better at predicting IC_80_ titers of triple combinations. Given this, we used the Bliss-Hill model to predict the coverage of scFv against a 45-virus panel at concentrations that correlated with protection in the AMP trials. Using IC_80_ titers and concentrations of 1μg/mL, there was 93% coverage for one dual scFv combination (3BNC117+10E8v4), and 96% coverage for two of the triple combinations (CAP256.25+3BNC117+10E8v4 and PGT121+3BNC117+10E8v4). Combinations of scFv, therefore, show significantly improved breadth and potency over individual scFv and given their size advantage, have potential for use in passive immunization.

## Introduction

Broadly neutralizing antibodies (bNAbs), isolated from a subset of HIV-1 positive individuals, are capable of neutralizing a wide range of HIV viruses. Crucially, bNAbs have been shown to provide protection in non-human primate studies and it is thought that such antibodies are needed for an effective HIV vaccine (1–4). However, to date, no candidate HIV vaccines have been able to elicit bNAbs in humans (5–8). This has led the field to actively explore the possibility of using bNAbs as biological drugs for passive immunization against HIV (9–13).

The results of the first efficacy trials of an antibody for HIV prevention tested in Africa and the Americas have recently been published (14–17). These two Antibody-Mediated Prevention (AMP www.ampstudy.org.za) trials showed that VRC01 had 75% prevention efficacy in high-risk men and women if the infecting virus was sensitive to the antibody at <1μg/ml (IC_80_). Therefore, to target the extensive envelope diversity, minimize escape, and provide sufficient potency a combination of multiple antibodies will be needed. Several studies have investigated the potential of antibody combinations and observed, as expected, an increase in breadth and potency (18, 19). These studies demonstrate that the complementary neutralization profiles of individual bNAbs can improve the overall breadth and provide higher coverage of multiclade panels of viruses at much lower antibody concentrations (18, 20). By using those antibodies that specifically target the HIV subtypes predominant in a specific area, a geographically relevant set of antibodies may be selected to provide optimal coverage and potency (20). For example, CAP256.25, which shows high potency against clade C viruses, is currently being assessed in combination with PGT121 and VRC07-523LS in dual and triple combination in the South African CAPRISA 012B trial (21, 22). Similarly, there are several ongoing phase 1 trials, testing multispecific antibodies or dual and triple combinations. These trials test the aforementioned antibodies in addition to V3 (10–1074) and V2 (PGDM1400) antibodies and the broadly neutralizing MPER-targeting antibody 10E8v4 (23, 24).

The ability to accurately predict the breadth and potency of antibody combinations without experimental validation enables the rapid identification of optimal combinations. Two models have been used to predict the IC_50_ and IC_80_ of antibody combinations based on single antibody titers, the Loewe Additive model, and the Bliss-Hill Independence model. Both models assume that there is no interaction between the different antibodies and that neutralization by combinations of antibodies will be additive, however, owing to different formulations of independence, their predicted results differ (18, 25). These models can also be used to determine whether synergy or antagonism occurs by comparing the predicted data with experimental results. When using the Loewe Additive model most combinations of two antibodies were demonstrated to show additive potency where the experimental potency was close to the predicted IC_50_ (19). This model in comparison to experimental results has in cases also indicated synergy or antagonism between antibodies targeting specific epitopes such as the CD4 binding site, MPER, and V1/V2 antibodies. However, comprehensive analyses have shown that the Bliss-Hill model tended to be better at predicting IgG combination titers (18, 19) and this model did not predict synergy between these epitopes when predictions were compared to experimental results.

Experimental synergy between anti-HIV antibodies has only been rarely observed, and only in the context of bispecific antibodies. A bispecific antibody that simultaneously engaged the V2 and V3 epitopes showed moderate levels of synergy (26). Another bispecific employing a CAP256.25 scFv and the antibody binding fragment (Fab) of 10-1074, showed moderate levels of improved potency against a few viruses (26). In this case, neutralization was compared to single scFv-Fc or IgG rather than to the predicted combination titers or experimental combinations of the two arms, which may have overestimated the level of synergy (20, 26–30). More convincing evidence of synergy was observed when antibodies targeting a host cell protein and the viral Env protein were combined particularly as part of bispecific or trispecific antibody constructs. This effect is due to the localization of the anti-HIV antibody close to the host cell membrane through CCR5 or CD4 binding, for example, the 10E8-iMab, which targets the MPER region on the HIV virion and the CD4 receptor on the HIV target cell. This bispecific antibody has a geometric mean IC_50_ potency of 0.002μg/mL compared to 0.4μg/mL for 10E8 and 0.05μg/mL for the iMab indicating a 25-fold improvement over the expected activity (27, 31).

Single chain variable fragments (scFv) are small molecules, which contain the variable heavy and light chain of antibodies connected through a glycine linker. These molecules have some enhanced pharmacokinetic properties such as improved distribution and absorption into mucosal tissues despite a loss of half-life due to lacking an Fc region (32–35). They may also display less steric hindrance when used in combination with other molecules and other bNAbs or scFv (36). This may be especially true for epitopes in close proximity such as the V2 and V3, or V3 and CD4bs. A recent study demonstrated that scFv targeting the V3 and CD4bs could display synergy when combined with fusion inhibitors (37).

We previously demonstrated that scFv of bNAbs retain significant breadth and potency against a multiclade panel of viruses despite potency differences linked to differential affinity for the epitope (38). In particular, 10E8v4 maintained the same breadth and most of its potency as an scFv. Single antibodies may be limited in their ability to prevent HIV infection, however, as evidenced by the recent AMP results. We, therefore, tested combinations of scFv that target major bNAb epitopes on the HIV trimer, namely CAP256.25 (V2 apex), PGT121 (N332-supersite), 3BNC117 (CD4bs), 8ANC195 (gp120-gp41), and 10E8v4 (MPER) and show that they generally follow a model of additive potency and complementary breadth. No significant antagonism or synergy was observed compared to the models, although antibody combinations tested against individual viruses could show variation. Overall, combinations of three scFv antibodies reached considerable breadth and potency indicating that scFv in combinations should be further investigated for passive immunity purposes.

## Methods

### scFv Construction

scFv were designed and cloned previously as described (38). In short, single constructs containing the variable heavy and light chain interspaced with a 15 or 18 amino acid glycine-serine linker of five HIV-directed bNAbs (CAP256.25, PGT121, 3BNC117, 8ANC195, and 10E8v4) were generated through overlapping PCR or ordered from GenScript (New Jersey, USA) (38). These scFv genes were then cloned into a CMV/R expression plasmid (AIDS Reagent Program, Division of AIDS, NIAID, NIH. For the lambda chain of PGT121, the pBR322 based lambda expression vector was used (AIDS Reagent Program, Division of AIDS, NIAID, NIH).

### scFv Protein Expression

The constructs were grown in JM109 bacterial cells and extracted using a plasmid Maxiprep kit (Qiagen, Hilden Germany). Sequences were confirmed using the Applied Biosystems 3500xL Genetic Analyzer. Constructs were expressed as previously described (39). In short, HEK293F suspension cells at 1.5x10^6^ to 2x10^6^ cells/ml were cotransfected with linear Polyethylenimine hydrochloride (molecular weight, 40,000) at a 3:1 ratio with 1μg of plasmid per 1 ml of culture. Supernatants were harvested after 6 days.

scFv proteins were purified using Ni-Sepharose beads (GE Healthcare, Massachusetts USA), washed using a 30mM imidazole–phosphate-buffered saline (PBS) solution and eluted using 400mM imidazole in PBS. Glycerol was added to the elution at a final concentration of 5% to limit aggregation. Eluates were applied to Hiload Superdex 75 or Superdex 200 columns (GE Healthcare) equilibrated with PBS at pH 6.5 (5% glycerol with 0.02% sodium azide). The fractions corresponding to the size of the scFv were collected, pooled, and concentrated using Vivaspin concentrators or Vivapore static concentrators (GE Healthcare). The samples were dialyzed overnight at room temperature to remove sodium azide. Concentrations were measured on a NanoDrop device (Thermofisher, MA, USA), with extinction coefficients at 1% calculated using Expasy ProtParam (40) and characterized by SDS-PAGE. Molar weight was determined by using Expasy ProtParam (CAP256.25: 31.35kDa, PGT121: 28.89kDa, 3BNC117 28.47kDa, 10E8v4: 29.30kDa and 8ANC195 29.00kDa). scFv proteins were stored at -75°C.

### IgG Production

IgG constructs were expressed in HEK293F cells as described previously (39). Supernatants were harvested after 6 days and purified using a protein A affinity column. Proteins were eluted using a 0.15M glycine buffer at pH 2.5 buffer into 1M Tris, pH 8, and were concentrated and dialyzed into PBS pH 6.5 containing 5% glycerol. Concentrations were measured on a Nanodrop using an Extinction Coefficient of 13.7 at a 1% solution. The molecular weight of the IgG was calculated using the Expasy ProtParam (40) of CAP256.25 (150.71kDa), 10E8v4 (147.29kDa), 3BNC117: (146.24kDa), PGT121: (146.2kDa), 8ANC195: (147.43kDa). IgG and proteins were stored at -75°C.

### Pseudovirus production for TZM-bl Assay

Plasmids containing HIV-1 envelope (gp160) genes cloned in the pcDNA™3.1D/V5-His-TOPO^®^ vector were co-transfected with pSG3^Δenv^ into HEK293T cells and cultured for 48-72hrs at 37°C. Supernatants were harvested and filtered through a 45μm filter and frozen at -80°C. Virus stocks were titrated on TZM-bl cells using a luciferase assay to determine a dilution yielding RLU at least 10-fold above the “cell only” background (40,000-100,000 RLU).

### Neutralization Assay

A panel of 43 viruses (41) plus BG505 N332 and CAP256_SU (CAP256.3mo.9C) (42, 43) representing HIV-1 clades A, B, and C was used to compare neutralization titers of IgG and scFv. Neutralization assays were performed in TZM-bl cells as described previously (44–46). Proteins were tested at 200μg/mL for the IgG (~146kDa) and 50μg/mL for the scFv (28-32 kDa). All assays were repeated at least twice. IC_50_ and IC_80_ of each antibody tested was calculated and geometric mean potency was calculated for both IgG and scFv using sensitive viruses only.

### Experimental Testing of scFv Combinations

Combinations were tested by adding equimolar amounts of two or three scFv proteins in a neutralization assay as described above. A panel of 17 subtype A, B and C viruses were selected based on their sensitivity to at least 2 of the scFv in order to test neutralization of scFv combinations and confirm the Loewe Additivity and Bliss-Hill Independence models. Pre-dilutions containing scFv at 2μM and 10μM each were used to facilitate the assay set up. As a control, the single scFv were diluted to 10μM and 2μM as well and run alongside the combinations as a comparison. The highest concentration tested for the combinations was 30μg/mL or 1000nM. IC_50_ and IC_80_ of each experimental antibody combination was calculated, where each antibody is present at the concentration determined at IC_50_ or IC_80._

### Loewe Additivity and Bliss-Hill Independence Models

The IC_50_ and IC_80_ values from experimental combinations were compared to the predicted IC_50_ and IC_80_ based on the Loewe Additive model and the Bliss-Hill model as previously described (18, 19).

The following formula is used to calculate Loewe Additivity.


$$Predicted{IC}_{50}=1/(\frac{1}{{IC}_{50}(A)}+\frac{1}{{IC}_{50}(B)}+\ldots \frac{1}{{IC}_{50}(N)})$$

Post analysis values were recalculated from nM into μg/mL based on the following formula.


$${IC}_{50}in\;\mu g/mL=\frac{\left(Mw(A)\times {IC}_{50}\;in\;nM)+(Mw(B)\times {IC}_{50}\;in\;nM)+\ldots (Mw(N)\times {IC}_{50}\;in\;nM\right)}{n\times 1000}$$

Where (n) is the number of antibodies, Mw the molecular weight of the antibodies in the combination, and IC_50_ the experimental IC_50_ or theoretical IC_50_ obtained. The same formula is used for IC_80_ values replacing the IC_50_ with IC_80_ in the formula above. For resistant viruses, the model assumes the titer of the active scFv.

For the Bliss-Hill Independence model, the following formula was used to calculate the Hill function


$$f(c)=\frac{c^{m}}{\left(k^{m}+c^{m}\right)}$$

Where c= bNAb concentration, k = IC_50_, and


$$m=\frac{\log(4)}{\log({IC}_{80})-\log({IC}_{50})}$$

The combination neutralization curve is then calculated using the Bliss Independence model,


f=1−(1−f(A))(1−f(B))(…)

with f(A), f(B), etc. being the individual functions of the scFv antibodies. Combination molar IC_50_ and IC_80_ titers are calculated by setting f = 0.5 or 0.8 and assuming each scFv is present at the same molarity, and converted to µg/ml using the above formula.

Dual/triple coverage was calculated by considering a virus resistant if less than 2/3 antibodies in the combination were able to neutralize that virus at set concentrations.

We used the following formula for both models to determine which is more accurate in predicting the combination potencies for IC_50_ and IC_80_.


$$Absolute(Log10(experimental\;{IC}_{50})-Log10(predicted\;{IC}_{50}))$$

### Synergy and/or Antagonism Predictions Based on Loewe Additivity and Bliss-Hill Independence Models

Synergy was predicted based on whether the experimental IC_50_ and IC_80_ were improved compared to the Loewe Additive model or the Bliss-Hill model. The formula below was used to characterize this effect, with the Bliss-Hill IC_50_ replacing the Loewe IC_50_ in the formula below.


$$LogFold=Log10(\frac{Loewe^{50}}{Experimental\;IC_{50}})$$

Positive values indicate experimental titers lower (i.e. more potent) than predicted and imply synergy, while negative values indicate less potent experimental titers than predicted and imply antagonism. For a given combination of antibodies, the mean and the 95% Confidence interval were calculated using LogFold values for this combination against each virus in the panel.

### Statistics

All statistics were done using the GraphPad Prism 8 software. Fold differences of the experimental combinations were calculated with the most potent scFv in the mixture for both IC_50_ and IC_80_. A Wilcoxon signed-rank test was performed to test which model was more accurate at predicting the experimental titers for both IC_50_ and IC_80_. Pearson’s Correlation between experimental neutralization titers and titers based on the two models was calculated using the GraphPad software. A nonlinear fit model (log-log) was used to predict the slope between the experimental and predicted data, using a robust regression. A log-fold with a 95% confidence interval was used to determine if the fold difference is significantly different from 0, indicating either synergy or antagonism. Breadth-potency curves were drawn using a survival model in GraphPad. Significance was tested using the log-rank Mantel-Cox significance test.

## Results

### Dual and Triple Combinations of scFv of HIV bNAbs

To assess whether scFv of HIV bNAbs showed increased breadth and potency when used in combination, we tested five different scFv as part of dual and triple combinations. The scFv included those targeting the V2 (CAP256.25), the N332 supersite (PGT121), the CD4bs (3BNC117), and the MPER region (10E8v4), all of which were previously shown to retain significant activity compared to IgG (38). For this study, we added the antibody 8ANC195 that targets the gp120-gp41 interface so that all five major epitopes on the HIV trimer were covered (see single IC_50_ and IC_80_ data in nM **Supplementary 1A** and **1B** and in µg/ml **1C** and **1D** respectively, (38)]. All scFv were expressed and purified by size exclusion columns and, with size and purity confirmed SDS-PAGE gels. scFv were stored in buffers containing 5% glycerol as determined previously to prevent aggregation (38).

**Figure 1:**
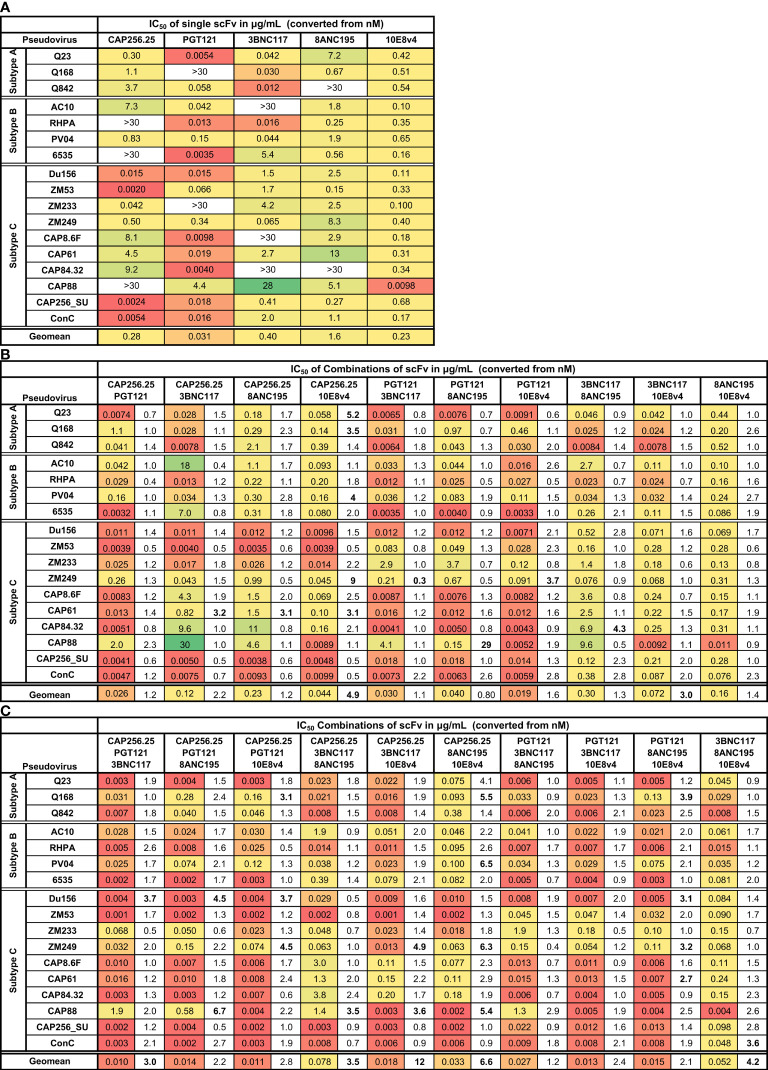
Neutralization titers of single, dual, and triple combinations of scFv. Heat map showing IC_50_ neutralization titers in μg/mL for single **(A)**, dual **(B)**, and triple **(C)** combinations of scFv against a panel of 17 subtype A, B, and C viruses. Viruses insensitive to individual bNAbs are shown as >30μg/mL. The fold improvement in IC_50_ titers of the dual and triple combinations relative to the IC_50_ of the best scFv in the combination is included in **(B, C)**. Values with >3-fold increase or decrease in neutralization are shown in bold. Geometric mean potency is included at the bottom of each table. Each scFv is present in the combination at the titer indicated.

Combinations of scFv were tested against a panel of 17 pseudoviruses from subtypes A, B, and C (n=3, 4 and 10, respectively). These were selected based on their sensitivity to at least three of the five scFv under investigation. Equimolar amounts (based on their molecular weights), of each scFv in combinations of two or three were tested, giving a total of 20 different combinations of antibodies (10 dual and 10 triple combinations), which were compared to single scFv neutralization titers. To standardize the output data, the concentration of the single scFv and the scFv combinations in μg/mL were calculated from the nM titers and the molecular weight of the scFvs (**Figure 1**, **Supplementary Figure 2**) (38). Combination titers IC_50_ and IC_80_ are reported as the concentration of each scFv in the mix in μg/mL or nM.

As expected, combinations of two scFv improved the coverage, with active scFv making up for the inactive scFv. For all dual and triple combinations, breadth reached 100% at IC_50_ against this 17-virus panel (**Figure 1**). The neutralization of individual viruses by two or three scFv was usually similar to the IC_50_ of the most potent scFv in the combination (**Figure 1**; fold differences are shown in the columns next to the IC_50_s). Except for a few cases, the virus neutralization titers of the dual and triple combinations fell within 3-fold of the titer of the most potent single scFv in that combination. There was little to no loss of potency noted compared to the most potent scFv for the entire panel for all 20 scFv combinations tested (a total of 340 single test titers). A few instances of potential gain of potency compared to the most potent scFv were noted, with most of these linked to specific scFv combinations (bolded values in **Figure 1**).(See **Supplementary Figure 2** for IC_50_ titers in nM **(A)** and μg/mL **(B)** and for IC_80_ values in nM **(C)** and μg/mL **(D)** respectively).

The combination of CAP256.25+10E8v4 scFv stood out, demonstrating an overall 4.9-fold improvement in geometric mean titer (**Figure 1B**). This was driven by five viruses of which two were subtype A, one was subtype B and two were subtype C indicating this was not subtype-specific. Five other dual scFv combinations showed improved potency over single scFv for single viruses but this was not linked to any specific combination of antibodies. The 3BNC117+10E8v4 combination showed an overall significant improvement in the geometric mean titer although this was not seen for individual viruses indicating complementarity of neutralization potency of the scFv rather than synergy.

For the triple combinations, five of the ten combinations showed >3-fold improved geometric means compared to the most potent single scFv with a 12-fold improvement for the CAP256.25+3BNC117+10E8v4 combination (**Figure 1C**). This was higher than for the dual combinations where only two out of ten showed an improvement in geometric mean potency and was driven by improvement in potency against single viruses (16 of the 170 virus-scFv combination pairings). This was due to the scFv (e.g. CAP256.25 and 3BNC117 scFv), in the combinations being potent against different viruses allowing for complementarity in neutralization potency and coverage. Triple combinations overall showed higher improvements in breadth compared to dual combinations or single scFv as a consequence of having more antibodies.

Some viruses appeared to be more sensitive to the effects of combined scFv. For example, a slight potency improvement was observed against the CAP61 and ZM249 viruses for the dual combinations, although for the latter loss of potency was also noted (fold difference <0.33 see **Figure 1B**). Some enhancement in potency was also seen with these viruses plus Du156 and CAP88 for the triple combinations (**Figure 1C**).

**Figure 2:**
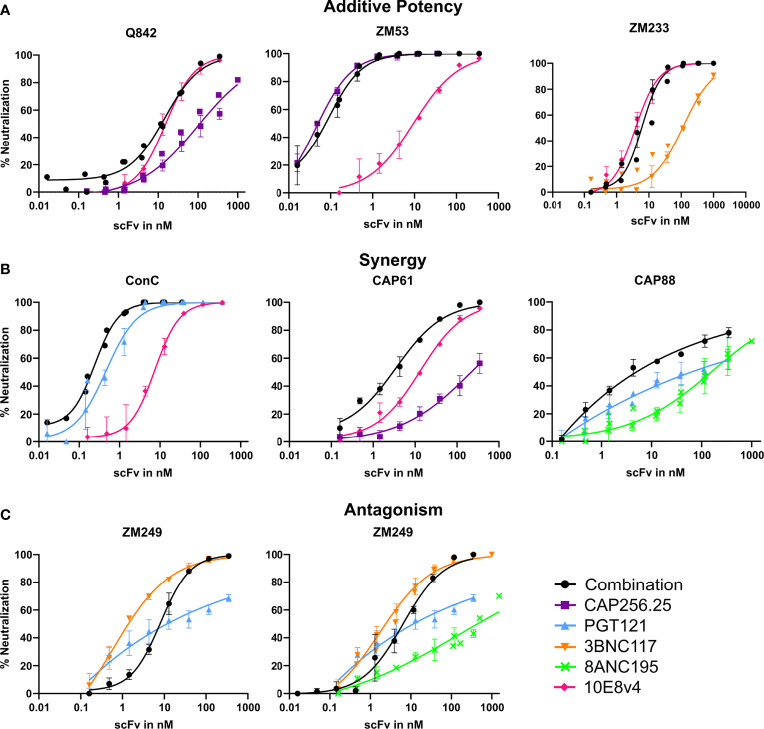
Combinations of scFv that show improved neutralization potency over single scFv. Neutralization curves showing potential additive, synergistic and antagonistic potency effects of combinations of antibodies. Combinations of scFv compared to single scFv neutralization curves with CAP256.25 in purple, PGT121 in light blue, 3BNC117 in orange, 8ANC195 in green and 10E8v4 in pink. The dual or triple combinations are represented in black. Geometric mean values are used for each data point with error bars representing repeat experiments. **(A)** Examples of combinations where neither synergy nor antagonism is observed for three viruses tested against dual combinations of scFv. Additive potency is represented by the combination curve (black) overlapping with the best scFv in the mixture. **(B)** Potential synergy as observed in dual combinations of scFv against three different virus strains. Synergy was represented by a left shift of the combination curve (black) relative to the most potent scFv in the mixture, or by steeper neutralization curves resulting in improved IC80. **(C)** Potential antagonism as represented by a right shift of the combination curve relative to the most potent scFv in the mixture, observed in combinations of two or three scFv tested against ZM249, which was sensitive to all scFv in the combinations.

Overall, we found either improved or similar titers for combinations compared to the most potent single scFv, particularly for triple combinations, indicating that combinations of scFv improve the coverage of a panel of viruses and the potency at which viruses are neutralized. We overlaid neutralization curves of the combinations with the single scFv used in the combination, to determine if there were distinct patterns associated with improved IC_50_s or where we observed similar IC_50_ for the combinations compared to the single scFv. For most dual and triple combinations (314/340), an additive effect was seen where the combination curve overlapped with the best scFv in the combination (**Figure 2A**). Here IC_50_ did not show a potency improvement compared to the single scFv. A few combinations shifted the curve to the left compared to the curve of the most potent single scFv corresponding with fold potency improvements as seen in **Figure 1** and indicating potential synergy (10/170 virus-dual combination pairings and 16/170 in the triple combinations showed improved IC_50_s) (**Figure 2B**). In one case we observed decreased potency for the combination compared to the most potent scFv possibly indicating some antagonism (**Figure 2C**). With the addition of a third antibody this was partially negated (**Figure 2C** right panel). Overall, most combinations did not show significant potency improvements or loss of potency, suggesting that they largely followed an additive model of potency.

### scFv Follow an Additive Model of Interaction in HIV Neutralization

To further explore whether combinations of scFv follow an additive model of interaction we compared the experimental results with predictions based on the single scFv titers using the Loewe Additive model and the Bliss-Hill models. As mentioned before, both these models assume no synergistic or antagonistic interactions between antibodies (18, 19). We calculated the combination scFv IC_50_ and IC_80_ titers based on these 2 models (Methods) and based on the geometric mean IC_50_ and IC_80_ of the repeats of single scFv obtained in the experiment. All scFv predicted data titers were compared to the IC_50_ and IC_80_ titers of the experimentally tested combinations.

**Figure 3:**
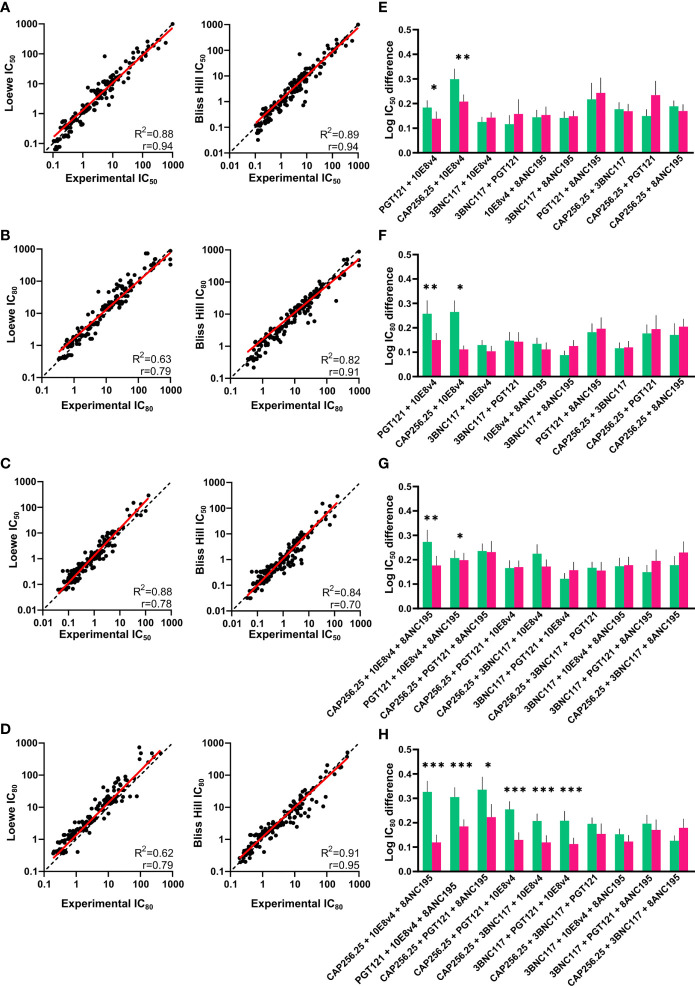
Comparison of experimental and predicted combinations of dual and triple scFv. **(A, B)** IC_50_ and IC_80_ titers of dual antibody combinations plotted against the predicted IC_50_ and IC_80_ titers according to the Loewe Additive (left) and Bliss-Hill Independence (right) models. **(C, D)** Predicted IC_50_ and IC_80_ titers of triple combinations *versus* the experimental IC_50_ and IC_80_ titers. Values where both or all titers of single scFv >30μg/mL are excluded. **(E, F)** Comparison of the absolute Log(IC_50_) and Log(IC_80_) difference between the experimental titers and predicted titers according to the Loewe Additive (green) and Bliss-Hill independence (purple) for the dual combinations. **(G, H)** Comparison of the absolute Log(IC_50_) and Log(IC_80_) difference between the experimental titers and predicted titers according to the Loewe Additive (green) and Bliss-Hill independence (purple) models for the triple combinations. For **(A, B, E, F)**, a nonlinear robust regression log-log line (red) and an equity line (black dotted) as well as r, R2, are shown and p<0.0001. For **(C, D, G, H)**, mean values are given with a standard error of the mean. A paired, Wilcoxon t-test was performed on each pair with significant p values above the graphs (p < 0.05 = * p < 0.01 = ** p < 0.001= ***).

There was a strong significant (p<0.0001) correlation between the experimental IC_50_ and IC_80_ and the predicted IC_50_ and IC_80_ of the dual combinations for both the Loewe and Bliss Hill model (Loewe, Pearson’s r=0.94 and r=0.79 respectively, Bliss-Hill, r=0.94 and 0.91 respectively) (**Figures 3A, B**). Similarly, for the triple combinations, there was a strong correlation between the predicted values and the experimental values (Loewe r=0.78 and r=0.79, Bliss-Hill: r=0.70 and r=0.95 respectively) (**Figures 3C, D**). For each combination, we also determined which model was better by comparing the mean absolute log difference between the experimental values and the predicted values for each individual combination. For the dual combinations, the Bliss Hill model was better at predicting two out of the 10 combinations (10E8v4 combined with CAP256.25 or PGT121), whereas no significant difference between the models was observed for any of the other combinations (**Figures 3E, F**). Similarly, for the triple combinations, most combinations did not show differences between the two models in the IC_50_ with the Bliss-Hill model being better at predicting two combinations (8ANC195 + 10E8v4 with either CAP256.25 or PGT121) (**Figure 3G**). For the IC_80_ of the triple combinations, 6/10 combinations were significantly better predicted by the Bliss-Hill model compared to the Loewe model (**Figure 3H**). This indicated that for most combinations both Loewe and Bliss-Hill could predict combination IC_50_ titers well with a slight advantage for the Bliss-Hill model. However, the Bliss-Hill model is significantly better at predicting triple IC_80_ titers compared to the Loewe model, with the latter underestimating the potency of the triple combinations at IC_80_.

### No Synergy or Antagonism Observed for scFv Combinations Against a Panel of HIV Pseudovirus

**Figure 4:**
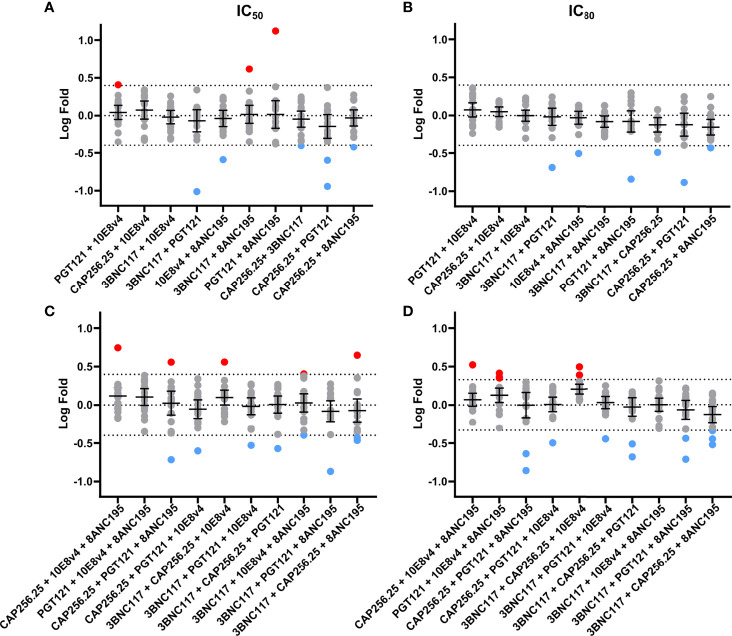
No synergy observed using fold difference between experimental and Bliss-Hill predictions of dual and triple scFv combinations. Log-fold differences of double and triple IC_50_ **(A, C)** and IC_80_ **(B, D)** experimental combinations to the Bliss-Hill Independence model prediction. Mean and the 95% confidence interval are shown with a dotted line indicating no fold change. Log (2.5) fold difference dotted lines (0.4 or -0.4) are also shown. Red and blue are used to indicate more than a log difference of 0.4 (~2.5x) with red dots indicating potential synergy and blue dots indicating potential antagonism.

We next sought to determine if any of the combinations showed significant potency improvement or potency loss in either IC_50_ or IC_80_ by comparing the experimental data to the expected titers based on the Bliss Hill model. These analyses allowed us to determine if there is potential synergy (higher potency than predicted), as indicated by a log-fold difference >0.4 (~2.5 fold) and/or antagonism (lower potency than predicted) as indicated by a log-fold difference <-0.4 (**Figure 4**). The dual combinations had a mean log-fold difference close to 0 (log-fold -0.4<CI<0.4) for both IC_50_ and IC_80_ indicating that there was neither antagonism nor synergy, although a few antibody combinations showed some improved potency (red dots, IC_50_ n=3, 1.7% and IC_80_: n=0, 0%) or decreased potency (blue dots, IC_50_ and IC_80_: n=6, 3.5%) for individual viruses these were relatively few and most showed less than 0.4 log fold difference when compared to model predictions (**Figures 4A, B**).

For the triple combinations, similar results were observed with 157/170 of the IC_50_ and 154/170 of the IC_80_ values showing less than a Log (2.5) difference. The mean on triple combinations was also close to 0 (log-fold -0.4<CI<0.4) for both IC_50_ and IC_80_. Similarly, only a few individual viral titers showed a potential loss of potency for the IC_50_ (n=8, 4.7%) and IC_80_ (n=11, 6.5%) or a potential gain of potency (IC_50_ and IC_80_: n=5, 3%) for triple combinations compared to the model (**Figures 4C, D**). None of the outliers in dual or triple combinations could be linked to specific viral signatures (data not shown). Notwithstanding these few outliers, we, did not observe any significant synergy nor antagonism for dual or triple combinations of scFv.

### Using the Bliss-Hill Model to Predict IC_50_ and IC_80_ Titers of scFv Combinations Against a Larger HIV Panel

**Figure 5:**
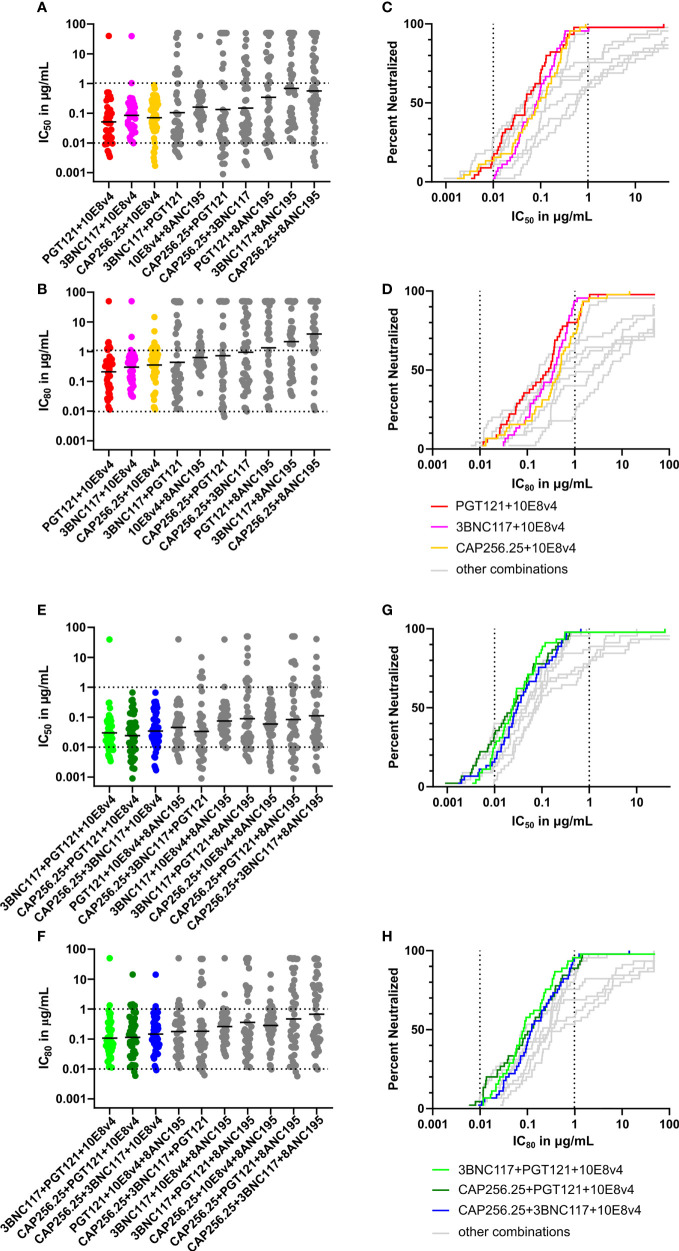
Predicted breadth and potency of scFv combinations against large virus panel. **(A)** Scatter plot showing predicted IC_50_ titers of dual combinations based on single scFv data for a panel of 45 viruses using the Bliss-Hill Independence model. The IC_50_ data is plotted in μg/mL recalculated from nM based on the formula given in the methods section. **(B)** Predicted IC_80_ titers of dual combinations based on the Bliss-Hill Independence model ranked by geometric mean. **(C)** Breadth-potency curves of IC_50_ titers of most potent and broad dual combinations were plotted for the IC_50_.with the others in grey. **(D)** Breadth-potency curves from IC_80_ data for the most potent and broad dual combinations. **(E)** IC_50_ titers for triple combinations based on the Bliss-Hill Independence model. **(F)** Predicted IC_80_ titers for the triple combinations ranked based on geometric mean. **(G)** Breadth potency curves for the IC_50_ of the most potent and broad triple combinations are plotted. **(H)** The breadth-potency curves are based on IC_80_ titers of the most potent and broad combinations. The maximum neutralization for the IC_50_ and IC_80_ data is set at 50μg/mL for all plots. Geometric mean is indicated by a black line in **A, C, E**, and **G** Dotted lines are shown at 0.01μg/mL and 1μg/mL for breadth-potency plots IC_50_ and the IC_80_ **(B, D, F, H)**. Each scFv is present in the combination at the titer indicated.

In order to assess their breadth-potency on a larger virus panel, we used the IC_50_ and IC_80_ data of single scFv to predict neutralization titers for combinations of two and three scFv, now including 8ANC195, on a 45-virus panel described in our previous study (38) (see **Supplementary Figure 1** for single IC_50_ in nM **(A)** and IC_80_ in nM **(B)** or μg/mL **(C, D)** titers) (38). These theoretical scFv combinations were ranked based on their geometric mean potency using both IC_50_ and IC_80_ data for dual and triple combinations (**Figures 5A, B, E, F**). Combinations with the expected highest breadth and potency were plotted using breadth-potency curves (**Figures 5C, D, G, H**).

The best dual combinations, namely PGT121+10E8v4 (red), 3BNC117+10E8v4 (pink), and CAP256.25+10E8v4 (orange) were able to neutralize most viruses barring one at IC_50_ and IC_80_ (**Figures 5A, B**). The breadth-potency curves of the three 10E8v4-containing combinations reached 100% neutralization at the IC_50_ and 98% neutralization at the IC_80_ (colored breadth-potency plots *versus* grey, **Figures 5C, D**) with most viruses neutralized between 0.1 and 1μg/mL at IC_80_. The fourth combination of 10E8v4 with 8ANC195 was less effective due to the low potency of both antibodies and the limited breadth of 8ANC195, [see **Supplementary Figure 3** (38)]. The other dual combinations showed reduced breadth (up to 30% at 50μg/mL) or reduced potency especially at IC_80_ (grey dots and lines). Some combinations such as PGT121+3BNC117 had a potent geometric mean below 0.1μg/mL at IC_50_, but there was a larger spread of neutralization titers and so breadth was lost at IC_80_ (**Figures 5A, B**). (See **Supplementary Figure 4** for IC_50_ in nM **(A)** and μg/mL **(B)** and IC_80_ in nM **(C)** and μg/mL **(D)** titers of dual, and triple combinations predicted by the Bliss-Hill Independence model).

The triple combinations showed higher levels of potency and complete neutralization with 8/10 combinations showing complete neutralization of the viral panel at IC_50_, and 6/10 showing near-complete neutralization at IC_80_ (**Figures 5E–H**). Moreover, 7/10 triple combinations had a geometric mean potency below 0.1μg/mL at IC_50_ (**Figure 5E**, green, dark green, and blue). Of these, three combinations all had a geometric mean IC_80_ below 1μg/mL and neutralized up to 96% of viruses (**Figures 5F, H**). This indicated that triple combinations are able to reach nearly 100% neutralization at lower concentrations for both IC_50_ and IC_80_ (see **Supplementary Figure 4** for IC_50_ in nM **(A)** and μg/mL **(B)** and IC_80_ in nM **(C)** and μg/mL **(D)** titers of dual, and triple combinations predicted by the Bliss-Hill Independence model).

**Figure 6:**
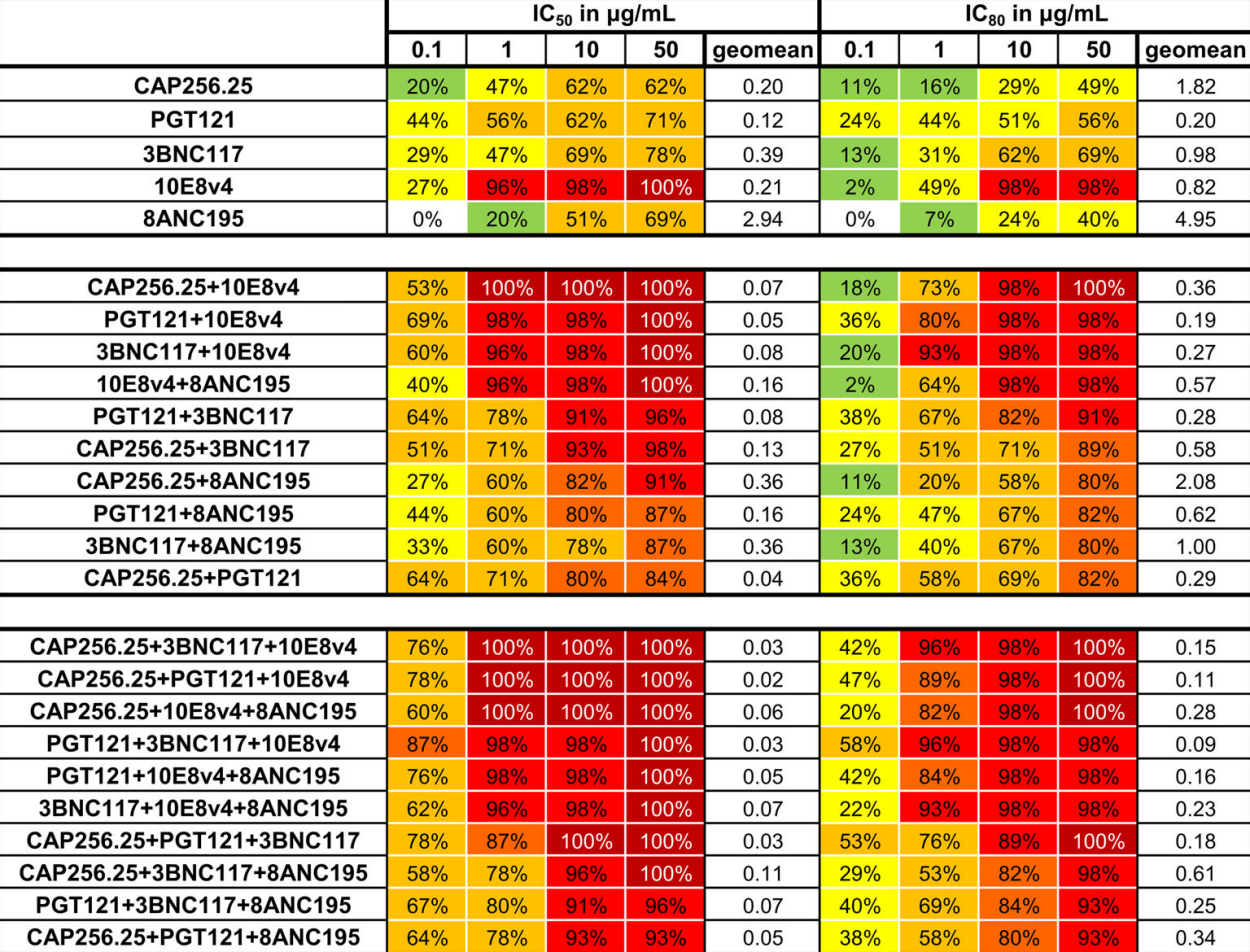
Neutralization breadth for single and combinations of scFv against a 45-virus panel at four concentrations. Percentage neutralization at 0.1, 1, 10, and 50μg/mL was calculated from single scFv titers and from predicted scFv dual and triple combination titers for the IC_50_ and IC_80_. Colors indicate percentage neutralization with 1-19% in green, 20-49% yellow, 50-79% yellow-orange, 80-89% orange, 90-99% red and 100% dark red. Geometric mean at 50μg/mL is also given. Each scFv is present in the combinations at the titers indicated.

The predicted breadth of each combination was next determined at four concentrations: 0.1, 1, 10, and 50μg/mL in addition to the geometric mean for the combinations tested (with a cutoff at 50μg/mL). Combining these scFv improved the coverage at all concentrations for both IC_50_ and IC_80_ (**Figure 6**). Breadth for dual combinations ranged from 84% to 100% at IC_50_ < 50µg/ml and 80% to 100% at IC_80_ < 50µg/ml. For dual combinations, 100% breadth was reached for one combination at IC_50_ < 1μg/mL, and for four combinations at IC_50_ < 50μg/mL. For the IC_80_, 98% breadth was obtained at 10μg/mL for the same combinations. Most combinations of three scFv had 100% breadth at IC_50_ < 50 µg/ml, except for PGT121+3BNC117+8ANC195 and CAP256.25+PGT121+8ANC195, which had a breadth of 96% and 93% respectively. Breadth was especially improved for lower concentrations at IC_80_. For example, breadth for single scFv ranged from 0 to 44% for IC_50_ < 0.1µg/ml and 0 to 24% for IC_80_ < 0.1µg/ml, whereas dual combinations ranged from 22 to 62% for IC_50_ and 2 to 29% for IC_80._ At the same cutoff, the breadth for combinations of 3 antibodies ranged from 47% to 82% for IC_50_ and 16 to 40% for IC_80_. Geometric mean potency for the panel also improved from 0.2-4.95 μg/mL for single scFv to 0.27-2.08μg/mL for dual combinations and 0.09-0.61μg/mL for triple combinations at IC_80_. This was expected as the titers were calculated as containing the same amount of each scFv at that concentration.

Since PGT121, 3BNC117, and CAP256.25 all had good potencies as scFv, using these together with 10E8v4 resulted in improved potency of the combination. Moreover, scFv such as CAP256.25 and PGT121 and/or 3BNC117 have complementary profiles, with 3BNC117, for example, being especially potent against subtype B viruses and CAP256.25 being potent against subtype C viruses (see **Supplementary Data 1** for the IC_50_ and IC_80_ of single scFv) (38). 10E8v4 is the broadest scFv and that resulted in the combinations containing 10E8v4 having the highest breadth. As a result, four of the five triple combinations with the best geometric mean titers included 10E8v4. Conversely, introducing less potent or less broad antibodies such as 8ANC195 resulted in less favorable antibody combinations, where 5 out of 6 combinations with the lowest potency and breadth contained 8ANC195, consistent with the relatively lower potency and breadth of this antibody. Of the top five, three combinations, CAP256.25+PGT121+10E8v4 and CAP256.25+3BNC117+10E8v4 and CAP256.25+8ANC195+10E8v4 neutralized all viruses below 1μg/mL and 98% of viruses at IC_80_ below 10μg/mL. The best ranked PGT121+3BNC117+10E8v4 combination neutralized 96% of viruses below 1μg/mL and 98% of viruses below 10μg/mL at the IC_80_ value. This analysis indicated a significant gain of potency and neutralization breadth at lower concentrations than single scFv at both IC_50_ and IC_80_. This indicated that combinations of scFv were able to increase the coverage at lower concentrations, which will be vital in clinical settings.

### Predicting the Efficacy of scFv Combinations

**Figure 7:**
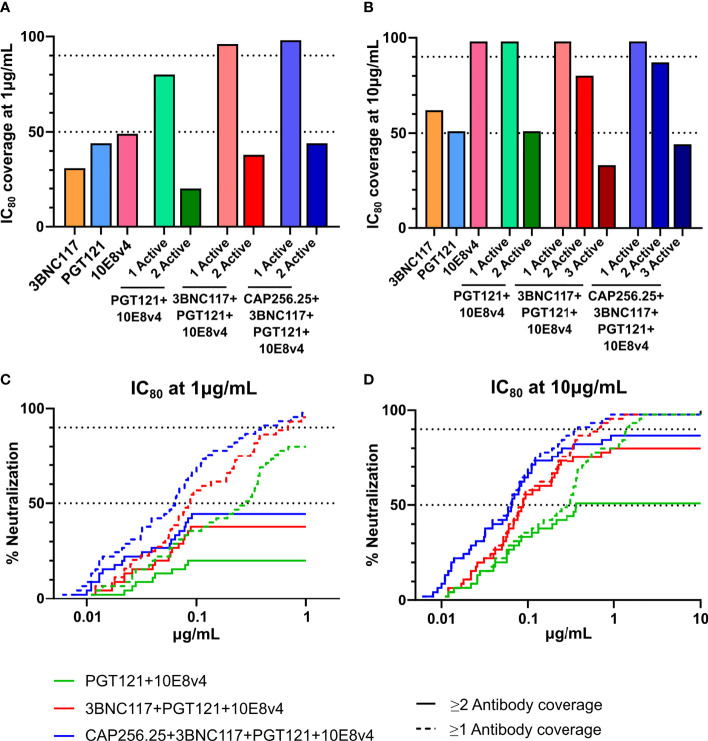
Predicted coverage and estimated efficacy of best scFv combinations. **(A, B)** Active coverage by one, two, three or four scFv was calculated at 2 concentrations (1μg/mL **(A)** and 10μg/mL **(B)** at IC_80_ based on the predicted titers according to the Bliss-Hill Independence model. The broadest triple and quadruple combinations are shown. **(C, D)** Breadth Potency curves of active coverage of 1 and 2 scFv are given for the best combinations (3BNC17+10E8v4 and 3BNC17+10E8v4+PGT121, and CAP256.25+3BNC17+10E8v4+PGT121) at 1 μg/mL **(C)** and 10μg/mL **(D)**. 50% and 90% breadth are indicated by the dotted lines in **(C, D)**.

The AMP trial showed that viral isolates that were sensitive to VRC01 with an IC_80_<1μg/mL were prevented from establishing infection in 75% of cases. As indicated in **Figures 5** and **6**, scFv combinations can reach significant coverage by a single antibody at this potency with the best scFv combination potentially reaching a 96% protection level. However, other antibody classes may need higher levels of potency to provide similar protection as VRC01 and viral isolates which are neutralized close to this cut-off may allow for breakthrough. Dual coverage will be able to overcome these issues by neutralizing breakthrough viruses at higher potencies and providing a back-up in case antibody levels drop. Therefore, using this cut-off, we calculated the predicted dual coverage (i.e. by at least two scFv) of the combinations using the thresholds of single scFv IC_80_ < 1µg/ml and < 10μg/mL. We found for dual combinations, the dual active coverage at 1μg/mL ranged from 20% with improvement at 10μg/mL to 51% for PGT121+10E8v4 (**Figures 7A, B**), This ranged from 0-20% at 1μg/mL and 9-62% for other combinations of two antibodies (See **Supplementary Figure 5**).

This was somewhat improved for triple combinations where we observed up to 58-80% dual active coverage at an IC_80_ of 10μg/mL. The best 3-combinations,3BNC117+PGT121+10E8v4 showing the highest active coverage at 10μg/mL of 80%. Generally, triple combinations showed higher levels of active coverage by 2 antibodies and often included both 10E8v4 and 3BNC117. Similar to the dual combinations, low dual coverage was observed at 1μg/mL up to 38% (See **Figure 7A**, **Supplementary Figure 5**. This could not be significantly improved for quadruple combinations which showed 44% and 87% dual coverage respectively at 1 and 10μg/mL. This loss of active coverage by multiple antibodies is also demonstrated in the breadth potency curves (**Figures 7C, D**). These curves clearly show that although at 10μg/mL most of the dual coverage is maintained there is a significant drop off at 1μg/mL. This was mainly due to antibodies such as 3BNC117, PGT121, and 10E8v4 showing only 31-49% breadth at 1μg/mL and the CAP256.25 scFv losing significant potency compared to the IgG (38). IgG combinations including this antibody could show slightly better active coverage by 2 antibodies, up to 53% at an IC_80_ of 1μg/mL and up to 91% at 10μg/mL (**Supplementary Data 6A** for predictions of IgG combinations titers in μg/mL and **6B** for neutralization breadth at 4 concentrations and dual coverage by IgG) (38). The other IgG and scFv showed similar levels of potency with specifically the 10E8v4 scFv retaining all of its potency explaining why the IgG combinations did not show higher levels of dual active coverage at 1μg/mL (**Figure 7**, **Supplementary Figure 6B**). This indicated that potency of single IgG and scFv needs to be improved to obtain higher levels of active coverage by 2 antibodies at lower antibody levels. The combination data demonstrate that the benefits of combinations of IgG targeting different HIV epitopes apply also to the smaller and more versatile scFv fragments.

## Discussion

Combinations of scFv antibodies were able to enhance the breadth and potency of HIV-1 neutralization compared to a single scFv. Specifically, several triple combinations reached 100% coverage at IC_50_ and IC_80_ due to complementary neutralization profiles. Dual and triple combinations of scFv demonstrated broad coverage in the potency range that correlated with prevention in the AMP trials (IC_80_<1μg/mL) and would therefore be expected to be highly effective in combating HIV transmission in high incidence areas.

Generally, dual scFv combinations followed an independent action model rather than a synergistic model of potency. The potency of the combination was usually similar to the potency of the most potent scFv in that mix. Although scFv may show less steric interference between antibodies this did not result in scFv showing significant potency improvements when used in combination. The close positioning of certain epitopes may prevent dual binding such as between V3 and CD4bs targeting scFv indicating that steric hindrance may have still impacted the scFv. Given the distinct MPER epitope located on the gp41 subunit instead of the gp120 subunit, we also expected independent effects for antibodies combined with 10E8v4, which was somewhat indicated in comparing experimental results with the most potent scFv. However, besides a few outliers, this was not apparent using the Bliss-Hill model. It is likely that binding of the MPER antibody confers conformational changes upon binding to its epitope precluding neutralization by other antibodies (47–50). scFv may be especially advantageous in bispecific constructs as their smaller size allows for easier expression. Bispecific products will also increase the local concentration of the second antibody and with the appropriate linker may in such cases allow for synergy. Similarly, minor losses of potency compared to the most potent scFv were overall a rare occurrence and were negated with the addition of a third antibody (in a triple combination with e.g. 10E8v4). The few cases of antagonism noted for scFv combinations may also be due to conformational changes rather than steric hindrance, which would occur after antibody binding. To explore these aspects, scFv-trimer complex crystallization studies would have to be done.

The Loewe model tended to underestimate the potency at IC_80_ particularly of triple combinations. It also often predicted low-level synergy for combinations containing the MPER antibody 10E8v4 and the V2 antibody CAP256.25. The Bliss-Hill model did not indicate significant antagonism and/or synergy for any combination at either IC_50_ or IC_80_. We did observe a few individual outliers where for example the combination showed some improvement in IC_50_ and IC_80_ (CAP256.25+3BNC117+10E8v4) or similarly (3BNC117+PGT121) showed loss of potency against individual viruses but no specific viral signature could be identified. Some of these may be limitations of the model itself as these tended to occur more often with viruses where the neutralization curves were not sigmoidal or with a low slope (18). The Bliss-Hill model tended to be better at predicting the IC_80_ titers and both models were matched for the IC_50_ titers. This finding is similar to IgG combinations where the Bliss-Hill model was also more accurate at predicting combination titers compared to the Loewe model, therefore the Bliss-Hill model was the preferred model for analyses of larger scFv datasets.

From a clinical perspective, the use of antibody combinations may mitigate the impact of suboptimal antibody levels. Overlapping neutralization can enhance coverage against single viruses, which ensures that complete viral neutralization is achieved at lower concentrations of antibody. The selection of antibody combinations with high potency (IC_80_) can more effectively counter declining antibody levels associated with passive administration. Although combinations of two scFv neutralized 100% of viruses at the highest concentration tested, the coverage was much reduced at lower concentrations, whereas triple antibody combinations demonstrated much higher coverage at lower concentrations by combining broad antibodies with very potent antibodies. For example, at an IC_50_ of 1μg/mL, 100% of viruses were neutralized and at an IC_80_ of 1μg/mL, 96% of viruses were neutralized. This demonstrates that complementary neutralization of the same virus is extremely advantageous, which was previously demonstrated for combinations of IgG (20).

Besides improved coverage, we also observed improved active coverage by 2 antibodies for triple combinations compared to dual combinations (19). In experimental combinations, neutralization slopes were also improved by combinations of antibodies, especially IC_80_ titers and neutralization plateaus (less than 100% neutralization of a virus) were also less common with antibody combinations (data not shown) (18). It may also be beneficial to use antibodies or scFv that bind trimers in different states; for example, CAP256.25 binds early, whereas 10E8v4 likely binds trimers that are in an intermediate state allowing for complementarity in virus neutralization (51–53). Clinically, active coverage by multiple antibodies will prevent antibody escape when the serum titers drop to close to the neutralization range. The AMP trial demonstrated that if the VRC01 antibody levels dropped, protection against less sensitive viruses (IC_80_> 1μg/mL) was lost (17). This may differ for other antibodies or for antibodies targeting different epitopes and combinations may also provide dual active coverage of the viral quasispecies further improving this level of protection (54, 55). This highlights the need to use more potent antibodies but also to improve individual antibody potency. In the future, bNAbs and scFv may also need to be assessed in PBMC neutralization assays which may more accurately resemble *in vivo* neutralization, and could more accurately predict potency (17, 55). We observed that although overall coverage was improved, active coverage by multiple antibodies was low for both IgG and scFv using this set of antibodies. There are multiple efforts ongoing to improve antibody potency and bioavailability. Titers are likely to improve if newer scFv are developed which retain better potency compared to the IgG. Moreover, the selection of antibodies with optimal potency to cover geographically relevant viruses will ensure efficacy in the region.

For long-term antibody-mediated protection, adherence, and maintaining sufficient plasma levels in a wider population is critically important. At lower concentrations, scFv and IgG lose coverage especially in the ability to fully neutralize the virus (concentrations below IC_80_ titers). This is also observed for IgG, indicating that all the antibodies in the combination must be kept at optimal levels. For IgG, one way to achieve this has been to introduce mutations in the Fc portion of the antibody to increase the half-life by up to 6 months (56, 57). A second option is to introduce them into the body *via* a stable expression system through a vectored immunoprophylaxis (VIP) approach. Some bNAbs were recently expressed using AAV, showing modest levels *in vivo* (58, 59). However, due to the space constraints on vectors, constructs with multi-specific modalities would prove difficult. Bispecific products, which would partially overcome this, are also limited by space constraints. scFv in comparison with their much smaller size may be more readily expressed on AAV, allowing for the expression of multiple scFv continuously. scFv have a genetic size approximately 2.6-times smaller than IgG allowing for the expression of minimally three scFv per AAV, and improved expression levels (9, 29, 60–66). AAV based scFv expression can help overcome the short half-life of scFv due to their lack of an Fc region. Some studies have shown success in the use of AAV based scFv for Alzheimer’s disease, and Amyotrophic Lateral Sclerosis amongst others (61, 67, 68). *In vivo* data will be required to determine the antigenicity and bioavailability of scFv. However, scFv conjugated to short amino acid peptides or linked to targeting molecules were shown to have significant increases in bioavailability and stability (69–72). These strategies mitigate some of the challenges facing scFv.

The scFv studied here demonstrated high breadth and potency when used in combination. Although only a limited number of scFv were tested on a small panel of viruses, we demonstrated their potential against a larger panel of viruses through modeling. By selecting bNAbs that target all five epitopes we also showed the potential for complementary effects. These scFv showed little to no antagonism. As more potent scFv are engineered combinations with even better efficacy may be found. In addition, as scFv are more readily expressed on vectors, this could in future provide, an alternative avenue for the use of bNAbs in passive immunization. The encouraging results of the AMP trials are expected to fast-track the need to consider long-term delivery approaches for those antibodies with clinical benefit.

## Data Availability Statement

The original contributions presented in the study are included in the article/**Supplementary Material**. Further inquiries can be directed to the corresponding author.

## Ethics Statement

Ethics approval was obtained as per local regulations (M160341). Antibody sequences were obtained from publicly available databases.

## Author Contributions

RD performed all experiments, data interpretation, figures and manuscript generation, and literature review. KW performed data analysis and advised on statistical analysis. PM and LM supervised the project and assisted with data interpretation and manuscript writing. All authors contributed to the article and approved the submitted version.

## Funding

We acknowledge research funding from the South African Medical Research Council (SAMRC) Flagship Project, the NIH through a U01 grant (U01AI116086), the Poliomyelitis Research Fund (PRF) through a PRF research grant (17/15), and the Centre for the AIDS Program of Research (CAPRISA). CAPRISA is funded by the South African HIV/AIDS Research and Innovation Platform of the South African Department of Science and Technology and was initially supported by the U.S. NIAID, NIH, U.S. Department of Health and Human Services grant U19 AI51794. R.T.V.D. is supported by a Poliomyelitis Research Foundation Ph.D. bursary (W 16/72). PM is supported by the South African Research Chairs Initiative of the Department of Science and Innovation and National Research Foundation of South Africa (grant no. 98341). KW is supported by the Bill and Melinda Gates Foundation Collaboration for AIDS Vaccine Discovery grant OPP1032144 (Comprehensive Antibody Vaccine Immune Monitoring Consortium CAVIMC) (https://www.cavd.org/Pages/default.aspx). The funders had no role in study design, data collection, and analysis, decision to publish, or preparation of the manuscript.

## Conflict of Interest

The authors declare that the research was conducted in the absence of any commercial or financial relationships that could be construed as a potential conflict of interest.

## Publisher’s Note

All claims expressed in this article are solely those of the authors and do not necessarily represent those of their affiliated organizations, or those of the publisher, the editors and the reviewers. Any product that may be evaluated in this article, or claim that may be made by its manufacturer, is not guaranteed or endorsed by the publisher.
