# Supplementary material for: Combinations of Single Chain Variable Fragments From HIV Broadly Neutralizing Antibodies Demonstrate High Potency and Breadth
Source: Front Immunol. 2021 Sep 16;12:734110. doi: 10.3389/fimmu.2021.734110 (PMC8481832; doi:10.3389/fimmu.2021.734110)
Supplement: Supplementary file 1 [file DataSheet_1.pdf]

Supplementary Figure 1

A

Pseudovirus	single scFv IC_{50} in nM				
	**CAP256.25**	**PGT121**	**3BNC117**	**10E8v4**	**8ANC195**
Q23.17	9.66	0.19	1.5	14.4	249.9
Q168.A2	34.93	>1595	1.1	17.3	22.9
Q259.D2.17	22.33	34.60	>1595	35.7	>1595
Q461.E2	28.71	>1595	1.8	17.3	276.2
Q769.D22	>1595	>1595	0.5	5.5	11.5
Q842.D12	116.74	2.00	0.4	18.6	1069.1
BG505.W6M 332N	4.54	0.56	0.6	3.4	9.1
CAAN5342.A2	>1595	2.29	22.7	29.3	>1595
AC10.0.29	233.03	1.46	>1595	3.5	62.7
RHPA4259.7	>1595	0.44	0.6	12.0	8.7
TRO.11	>1595	2.23	1.6	8.9	7.0
PVO.4	26.55	5.34	1.5	22.3	66.4
SC422661.8	>1595	105.45	3.5	1.7	115.8
REJO.67	>1595	>1595	9.3	4.5	39.1
TRJO4551.18	>1595	830.45	91.5	13.2	491.4
WITO.33	>1595	>1595	0.9	4.4	>1595
THRO4156.18	>1595	>1595	70.3	4.2	>1595
QH0692.42	>1595	>1595	3.6	12.0	233.9
6535.3	>1595	0.12	188.0	5.6	19.4
Du151.2	1.10	0.17	1053.7	9.0	>1595
Du156.12	0.47	0.51	52.0	1.3	86.1
Du172.17	>1595	337.88	>1595	1.8	1369.9
Du422.1	8.85	7.72	>1595	4.4	>1595
ZM53M.PB12	0.06	2.28	60.5	11.1	5.3
ZM109F.PB4	35.09	>1595	>1595	3.4	>1595
ZM135M.PL10A	>1595	>1595	1.8	2.2	>1595
ZM197M.PB7	0.32	>1595	17.5	1.0	1369.9
ZM214M.PL15	16.08	1.51	6.2	4.6	34.5
ZM233M.PB6	0.99	588.24	108.2	3.4	85.1
ZM249M.PL1	16.08	11.68	2.3	13.7	285.8
CAP8.6F	257.00	0.34	>1595	6.0	100.7
CAP45.2.00.G3	3.39	1211.07	>1595	3.6	804.7
CAP61.4.22.F10A	144.00	0.67	93.1	10.7	458.3
CAP63.A9	4.90	5.32	21.1	17.5	>1595
CAP84.32	293.00	0.14	562.0	11.5	>1595
CAP85.9	>1595	1.83	597.1	2.9	1207.1
CAP88.B5	>1595	153.33	998.9	0.3	176.9
CAP206.8	4.51	692.04	>1595	2.7	>1595
CAP210.2.00.E8	0.11	>1595	14.5	11.9	>1595
CAP228.51	24.93	>1595	>1595	1370.0	>1595
CAP239.2.00.G3J	2.91	0.77	>1595	3.2	>1595
CAP244.D3	>1595	>1595	70.2	9.2	193.7
CAP255.2.00.16J	>1595	0.35	35.1	13.6	862.2
CAP256.SU	0.08	0.64	14.5	23.1	9.4
ConC	0.17	0.56	70.8	5.9	37.4
**Geometric mean**	**6.5**	**4.0**	**13.6**	**7.2**	**101.3**
**Breadth**	**62%**	**71%**	**78%**	**100%**	**69%**

B

Pseudovirus	single scFv IC_{80} in nM				
	**CAP256.25**	**PGT121**	**3BNC117**	**10E8v4**	**8ANC195**
Q23.17	350.00	0.50	5.1	52.9	593.1
Q168.A2	>1595	>1595	3.4	52.0	89.9
Q259.D2.17	1000.00	>1595	>1595	108.4	>1595
Q461.E2	223.81	>1595	6.3	71.4	>1595
Q769.D22	>1595	>1595	1.4	46.5	86.5
Q842.D12	1218.50	13.03	1.1	68.7	>1595
BG505.W6M 332N	67.54	1.16	1.6	14.8	45.1
CAAN5342.A2	>1595	36.23	63.5	160.0	>1595
AC10.0.29	>1595	8.24	>1595	13.0	>1595
RHPA4259.7	>1595	2.36	2.1	39.5	36.1
TRO.11	>1595	7.33	6.2	37.8	29.9
PVO.4	324.90	24.22	4.7	87.2	326.1
SC422661.8	>1595	536.41	9.9	10.1	>1595
REJO.67	>1595	>1595	42.8	16.5	308.9
TRJO4551.18	>1595	>1595	346.1	46.2	>1595
WITO.33	>1595	>1595	3.1	17.3	>1595
THRO4156.18	>1595	>1595	214.3	12.9	>1595
QH0692.42	>1595	>1595	9.9	48.5	>1595
6535.3	>1595	0.41	327.0	19.2	103.8
Du151.2	51.53	0.99	>1595	27.0	>1595
Du156.12	4.58	1.10	281.3	6.6	346.5
Du172.17	>1595	>1595	>1595	14.5	>1595
Du422.1	487.75	137.41	>1595	14.6	>1595
ZM53M.PB12	0.43	148.92	378.9	68.9	28.0
ZM109F.PB4	>1595	>1595	>1595	23.8	>1595
ZM135M.PL10A	>1595	>1595	6.2	11.0	>1595
ZM197M.PB7	3.19	>1595	64.8	5.2	>1595
ZM214M.PL15	716.09	20.28	73.0	52.6	>1595
ZM233M.PB6	22.54	>1595	490.1	15.4	878.5
ZM249M.PL1	1594.90	732.15	16.0	80.3	1000.0
CAP8.6F	>1595	2.00	>1595	22.3	364.3
CAP45.2.00.G3	683.66	>1595	>1595	11.9	>1595
CAP61.4.22.F10A	978.48	3.60	251.6	60.1	>1595
CAP63.A9	133.98	26.57	113.8	70.8	>1595
CAP84.32	>1595	0.41	1622.6	34.6	>1595
CAP85.9	>1595	6.15	>1595	16.3	>1595
CAP88.B5	>1595	>1595	>1595	1.4	481.9
CAP206.8	69.47	>1595	>1595	11.0	>1595
CAP210.2.00.E8	0.49	>1595	>1595	68.1	>1595
CAP228.51	>1595	>1595	>1595	>1595	>1595
CAP239.2.00.G3J	34.16	5.88	>1595	16.8	>1595
CAP244.D3	>1595	>1595	340.9	39.4	536.5
CAP255.2.00.16J	>1595	0.98	242.6	66.5	>1595
CAP256.SU	0.45	2.11	56.7	86.5	31.5
ConC	1.61	1.63	233.2	19.2	252.6
**Geometric mean**	**58.0**	**6.8**	**34.4**	**28.0**	**170.6**
**Breadth**	**49%**	**56%**	**69%**	**98%**	**40%**

C

Pseudovirus	single scFv IC_{50} in µg/mL				
	**CAP256.25**	**PGT121**	**3BNC117**	**10E8v4**	**8ANC195**
Q23.17	0.30	0.0054	0.042	0.42	7.2
Q168.A2	1.1	>50	0.030	0.51	0.67
Q259.D2.17	0.70	1.00	>50	1.0	>50
Q461.E2	0.90	>50	0.050	0.51	8.0
Q769.D22	>50	>50	0.014	0.16	0.33
Q842.D12	3.7	0.058	0.012	0.54	31
BG505.W6M 332N	0.14	0.016	0.016	0.098	0.26
CAAN5342.A2	>50	0.066	0.65	0.86	>50
AC10.0.29	7.3	0.042	>50	0.10	1.8
RHPA4259.7	>50	0.013	0.016	0.35	0.25
TRO.11	>50	0.064	0.047	0.26	0.20
PVO.4	0.83	0.15	0.044	0.65	1.9
SC422661.8	>50	3.0	0.099	0.050	3.4
REJO.67	>50	>50	0.27	0.13	1.1
TRJO4551.18	>50	24	2.6	0.39	14
WITO.33	>50	>50	0.025	0.13	>50
THRO4156.18	>50	>50	2.0	0.12	>50
QH0692.42	>50	>50	0.10	0.35	6.8
6535.3	>50	0.0035	5.4	0.16	0.56
Du151.2	0.034	0.0049	30	0.26	>50
Du156.12	0.015	0.015	1.5	0.039	2.5
Du172.17	>50	9.8	>50	0.052	40
Du422.1	0.28	0.22	>50	0.13	>50
ZM53M.PB12	0.0020	0.066	1.7	0.33	0.15
ZM109F.PB4	1.1	>50	>50	0.098	>50
ZM135M.PL10A	>50	>50	0.050	0.063	>50
ZM197M.PB7	0.0100	>50	0.50	0.029	40
ZM214M.PL15	0.50	0.043	0.18	0.14	1.00
ZM233M.PB6	0.031	17	3.1	0.100	2.5
ZM249M.PL1	0.50	0.34	0.065	0.40	8.3
CAP8.6F	8.1	0.0098	>50	0.18	2.9
CAP45.2.00.G3	0.11	35	>50	0.10	23
CAP61.4.22.F10A	4.5	0.019	2.7	0.31	13
CAP63.A9	0.15	0.15	0.60	0.51	>50
CAP84.32	9.2	0.0040	16	0.34	>50
CAP85.9	>50	0.053	17	0.085	35
CAP88.B5	>50	4.4	28	0.0098	5.1
CAP206.8	0.14	20	>50	0.078	>50
CAP210.2.00.E8	0.0034	>50	0.41	0.35	>50
CAP228.51	0.78	>50	>50	40	>50
CAP239.2.00.G3J	0.091	0.022	>50	0.095	>50
CAP244.D3	>50	>50	2.0	0.27	5.6
CAP255.2.00.16J	>50	0.0100	1.0	0.40	25
CAP256.SU	0.0024	0.018	0.41	0.68	0.27
ConC	0.0054	0.016	2.0	0.17	1.1
**Geometric mean**	**0.20**	**0.12**	**0.39**	**0.21**	**2.94**
**Breadth**	**62%**	**71%**	**78%**	**100%**	**69%**

D

Pseudovirus	single scFv IC_{80} in µg/mL				
	**CAP256.25**	**PGT121**	**3BNC117**	**10E8v4**	**8ANC195**
Q23.17	11	0.014	0.15	1.5	17
Q168.A2	>50	>50	0.097	1.5	2.6
Q259.D2.17	31	>50	>50	3.2	>50
Q461.E2	7.0	>50	0.18	2.1	>50
Q769.D22	>50	>50	0.041	1.4	2.5
Q842.D12	38	0.38	0.032	2.0	>50
BG505.W6M 332N	2.1	0.034	0.047	0.43	1.3
CAAN5342.A2	>50	1.0	1.8	4.7	>50
AC10.0.29	>50	0.24	>50	0.38	>50
RHPA4259.7	>50	0.068	0.060	1.2	1.0
TRO.11	>50	0.21	0.18	1.1	0.87
PVO.4	10	0.70	0.13	2.6	9.5
SC422661.8	>50	15	0.28	0.30	>50
REJO.67	>50	>50	1.2	0.48	9.0
TRJO4551.18	>50	>50	9.9	1.4	>50
WITO.33	>50	>50	0.088	0.51	>50
THRO4156.18	>50	>50	6.1	0.38	>50
QH0692.42	>50	>50	0.28	1.4	>50
6535.3	>50	0.012	9.3	0.56	3.0
Du151.2	1.6	0.028	>50	0.79	>50
Du156.12	0.14	0.032	8.0	0.19	10
Du172.17	>50	>50	>50	0.42	>50
Du422.1	15	4.0	>50	0.43	>50
ZM53M.PB12	0.013	4.3	11	2.0	0.81
ZM109F.PB4	>50	>50	>50	0.70	>50
ZM135M.PL10A	>50	>50	0.18	0.32	>50
ZM197M.PB7	0.100	>50	1.8	0.15	>50
ZM214M.PL15	22	0.59	2.1	1.5	>50
ZM233M.PB6	0.71	>50	14	0.45	25
ZM249M.PL1	50	21	0.45	2.4	29
CAP8.6F	>50	0.058	>50	0.65	11
CAP45.2.00.G3	21	>50	>50	0.35	>50
CAP61.4.22.F10A	31	0.10	7.2	1.8	>50
CAP63.A9	4.2	0.77	3.2	2.1	>50
CAP84.32	>50	0.012	46	1.0	>50
CAP85.9	>50	0.18	>50	0.48	>50
CAP88.B5	>50	>50	>50	0.040	14
CAP206.8	2.2	>50	>50	0.32	>50
CAP210.2.00.E8	0.015	>50	>50	2.0	>50
CAP228.51	>50	>50	>50	>50	>50
CAP239.2.00.G3J	1.1	0.17	>50	0.49	>50
CAP244.D3	>50	>50	9.7	1.2	16
CAP255.2.00.16J	>50	0.028	6.9	1.9	>50
CAP256.SU	0.014	0.061	1.6	2.5	0.91
ConC	0.050	0.047	6.6	0.56	7.3
**Geometric mean**	**1.8**	**0.20**	**1.0**	**0.82**	**4.9**
**Breadth**	**49%**	**56%**	**69%**	**98%**	**40%**

Supplementary Figure 2

A

Experimental IC_{50} titres in nM

Pseudovirus	single scFv					2 scFv combinations										3 scFv combinations									
	CAP256.25	PGT121	3BNC117	10E8v4	8ANC195	CAP256.25 +PGT121	CAP256.25 +3BNC117	CAP256.25 +10E8v4	CAP256.25 +8ANC195	PGT121 +3BNC117	PGT121 +10E8v4	PGT121 +8ANC195	3BNC117 +8ANC195	3BNC117 +10E8v4	10E8v4 +8ANC195	CAP256.25 +PGT121 +10E8v4	CAP256.25 3BNC117 +10E8v4	CAP256.25 +10E8v4 +8ANC195	PGT121 +3BNC117 +10E8v4	PGT121 +10E8v4 +8ANC195	3BNC117 +10E8v4 +8ANC195	CAP256.25 +3BNC117 +PGT121	CAP256.25 +PGT121 8ANC195	CAP256.25 +3BNC117 +8ANC195	3BNC117 +PGT121 +8ANC195
Q23.17	9.7	0.19	1.5	14	250	0.24	0.94	1.9	5.9	0.23	0.31	0.26	1.6	1.5	15	0.10	0.75	2.5	0.18	0.16	1.6	0.098	0.13	0.79	0.20
Q168.A2	35	>1000	1.1	17	23	37	0.93	4.7	9.8	1.1	16	34	0.88	0.84	6.7	5.4	0.54	3.1	0.81	4.5	1.0	1.1	9.3	0.70	1.2
Q842.D12	117	2.0	0.41	19	>1000	1.4	0.26	13	71	0.22	1.0	1.5	0.29	0.27	18	1.5	0.27	13	0.19	0.78	0.27	0.22	1.3	0.26	0.21
AC10.0.29	233	1.5	>1000	3.5	63	1.4	592	3.1	36	1.2	0.55	1.5	93	3.7	3.4	1.0	1.7	1.5	0.78	0.73	2.1	0.93	0.81	66	1.4
RHPA4259.7	>1000	0.44	0.56	12	8.7	0.98	0.43	6.5	7.3	0.40	0.94	0.88	0.80	0.82	5.5	0.84	0.36	3.2	0.25	0.21	0.51	0.17	0.26	0.49	0.25
PVO.4	27	5.3	1.5	22	66	5.4	1.1	5.3	9.8	1.3	3.6	2.9	1.2	1.1	8.4	3.9	0.77	3.3	1.0	2.6	1.2	0.85	2.5	1.3	1.2
6535.3	>1000	0.12	188	5.6	19	0.11	233	2.6	10	0.12	0.11	0.14	9.1	3.9	3.0	0.11	2.7	2.7	0.13	0.11	2.8	0.070	0.069	13	0.17
Du156.12	0.47	0.51	52	1.3	86	0.35	0.36	0.32	0.40	0.43	0.24	0.42	18	2.5	2.4	0.14	0.31	0.34	0.25	0.17	2.9	0.13	0.11	0.98	0.26
ZM53M.PB12	0.064	2.3	60	11	5.3	0.13	0.13	0.13	0.12	2.9	0.98	1.7	5.4	9.5	9.5	0.054	0.050	0.054	1.6	1.1	3.1	0.040	0.051	0.082	1.6
ZM233M.PB6	0.99	>1000	108	3.4	85	0.83	0.57	0.47	0.86	103	4.1	126	48	6.2	4.3	0.78	0.76	0.59	6.4	3.6	5.2	2.3	1.7	1.6	24
ZM249M.PL1	16	12	2.3	14	286	8.7	1.4	1.5	33	7.4	3.1	23	2.7	2.3	11	2.5	0.45	2.1	1.9	3.7	2.3	1.1	5.1	2.1	5.3
CAP8.6F	257	0.34	>1000	6.0	101	0.28	145	2.3	49	0.30	0.28	0.26	125	8.2	5.3	0.20	3.8	2.6	0.37	0.21	3.9	0.35	0.22	101	0.46
CAP61.4.22.F10A	144	0.67	93	11	458	0.45	27	3.3	48	0.57	0.42	0.42	87	7.5	5.8	0.27	4.9	3.6	0.44	0.24	8.5	0.55	0.35	46	0.53
CAP84.32	293	0.14	>1000	11	>1000	0.17	323	5.2	365	0.14	0.15	0.17	241	8.7	11	0.23	6.6	5.9	0.14	0.16	5.1	0.11	0.11	130	0.21
CAP88.B5	>1000	153	999	0.34	177	65	1000	0.29	154	141	0.18	5.3	335	0.32	0.38	0.15	0.093	0.060	0.18	0.14	0.13	66	20	34	46
CAP256.SU	0.077	0.64	15	23	9.4	0.14	0.17	0.16	0.13	0.63	0.48	0.62	4.1	7.3	9.7	0.079	0.10	0.081	0.40	0.45	3.4	0.066	0.15	0.090	0.75
ConC	0.17	0.56	71	5.9	37	0.15	0.25	0.33	0.31	0.26	0.20	0.22	13	3.0	2.6	0.097	0.21	0.19	0.27	0.29	1.7	0.089	0.067	0.28	0.32
**Geometric mean**	**8.8**	**1.1**	**14**	**7.4**	**55**	**0.87**	**4.2**	**1.4**	**7.5**	**1.0**	**0.66**	**1.4**	**10**	**2.5**	**5.4**	**0.38**	**0.60**	**1.1**	**0.46**	**0.51**	**1.8**	**0.36**	**0.49**	**2.6**	**0.87**

B

Experimental IC_{50} titres in µg/mL

Pseudovirus	single scFv					2 scFv combinations										3 scFv combinations									
	CAP256.25	PGT121	3BNC117	10E8v4	8ANC195	CAP256.25 +PGT121	CAP256.25 +3BNC117	CAP256.25 +10E8v4	CAP256.25 +8ANC195	PGT121 +3BNC117	PGT121 +10E8v4	PGT121 +8ANC195	3BNC117 +8ANC195	3BNC117 +10E8v4	10E8v4 +8ANC195	CAP256.25 +PGT121 +10E8v4	CAP256.25 3BNC117 +10E8v4	CAP256.25 +10E8v4 +8ANC195	PGT121 +3BNC117 +10E8v4	PGT121 +10E8v4 +8ANC195	3BNC117 +10E8v4 +8ANC195	CAP256.25 +3BNC117 +PGT121	CAP256.25 +PGT121 8ANC195	CAP256.25 +3BNC117 +8ANC195	3BNC117 +PGT121 +8ANC195
Q23.17	0.30	0.0054	0.042	0.42	7.2	0.0074	0.028	0.058	0.18	0.0065	0.0091	0.0076	0.046	0.042	0.44	0.003	0.022	0.075	0.005	0.005	0.045	0.003	0.004	0.023	0.006
Q168.A2	1.1	>30	0.030	0.51	0.67	1.1	0.028	0.14	0.29	0.031	0.46	0.97	0.025	0.024	0.20	0.16	0.016	0.093	0.023	0.13	0.029	0.031	0.28	0.021	0.033
Q842.D12	3.7	0.058	0.012	0.54	>30	0.041	0.0078	0.39	2.1	0.0064	0.030	0.043	0.0084	0.0078	0.52	0.046	0.008	0.38	0.006	0.023	0.008	0.007	0.040	0.008	0.006
AC10.0.29	7.3	0.042	>30	0.10	1.8	0.042	18	0.093	1.1	0.033	0.016	0.044	2.7	0.11	0.10	0.030	0.051	0.046	0.022	0.021	0.061	0.028	0.024	1.9	0.041
RHPA4259.7	>30	0.013	0.016	0.35	0.25	0.029	0.013	0.20	0.22	0.012	0.027	0.025	0.023	0.024	0.16	0.025	0.011	0.095	0.007	0.006	0.015	0.005	0.008	0.014	0.007
PVO.4	0.83	0.15	0.044	0.65	1.9	0.16	0.034	0.16	0.30	0.036	0.11	0.083	0.034	0.032	0.24	0.12	0.023	0.100	0.029	0.075	0.035	0.025	0.074	0.038	0.034
6535.3	>30	0.0035	5.4	0.16	0.56	0.0032	7.0	0.080	0.31	0.0035	0.0033	0.0040	0.26	0.11	0.086	0.003	0.079	0.082	0.004	0.003	0.081	0.002	0.002	0.39	0.005
Du156.12	0.015	0.015	1.5	0.039	2.5	0.011	0.011	0.0096	0.012	0.012	0.0071	0.012	0.52	0.071	0.069	0.004	0.009	0.010	0.007	0.005	0.084	0.004	0.003	0.029	0.008
ZM53M.PB12	0.0020	0.066	1.7	0.33	0.15	0.0039	0.0040	0.0039	0.0035	0.083	0.028	0.049	0.16	0.28	0.28	0.002	0.001	0.002	0.047	0.032	0.090	0.001	0.002	0.002	0.045
ZM233M.PB6	0.031	>30	3.1	0.100	2.5	0.025	0.017	0.014	0.026	2.9	0.12	3.7	1.4	0.18	0.13	0.023	0.023	0.018	0.18	0.10	0.15	0.068	0.050	0.048	0.70
ZM249M.PL1	0.50	0.34	0.065	0.40	8.3	0.26	0.043	0.045	0.99	0.21	0.091	0.67	0.076	0.068	0.31	0.074	0.013	0.063	0.054	0.11	0.068	0.032	0.15	0.063	0.15
CAP8.6F	8.1	0.0098	>30	0.18	2.9	0.0083	4.3	0.069	1.5	0.0087	0.0082	0.0076	3.6	0.24	0.15	0.006	0.11	0.077	0.011	0.006	0.11	0.010	0.007	3.0	0.013
CAP61.4.22.F10A	4.5	0.019	2.7	0.31	13	0.013	0.82	0.10	1.5	0.016	0.012	0.012	2.5	0.22	0.17	0.008	0.15	0.11	0.013	0.007	0.24	0.016	0.010	1.3	0.015
CAP84.32	9.2	0.0040	>30	0.34	>30	0.0051	9.6	0.16	11	0.0041	0.0043	0.0050	6.9	0.25	0.31	0.007	0.20	0.18	0.004	0.005	0.15	0.003	0.003	3.8	0.006
CAP88.B5	>30	4.4	28	0.0098	5.1	2.0	30	0.0089	4.6	4.1	0.0052	0.15	9.6	0.0092	0.011	0.004	0.003	0.002	0.005	0.004	0.004	1.9	0.58	1.0	1.3
CAP256.SU	0.0024	0.018	0.41	0.68	0.27	0.0041	0.0050	0.0048	0.0038	0.018	0.014	0.018	0.12	0.21	0.28	0.002	0.003	0.002	0.012	0.013	0.098	0.002	0.004	0.003	0.022
ConC	0.0054	0.016	2.0	0.17	1.1	0.0047	0.0075	0.0099	0.0093	0.0073	0.0059	0.0063	0.38	0.087	0.076	0.003	0.006	0.006	0.008	0.008	0.048	0.003	0.002	0.008	0.009
**Geometric mean**	**0.28**	**0.031**	**0.39**	**0.22**	**1.6**	**0.026**	**0.12**	**0.044**	**0.23**	**0.030**	**0.019**	**0.040**	**0.30**	**0.072**	**0.16**	**0.011**	**0.018**	**0.033**	**0.013**	**0.015**	**0.052**	**0.010**	**0.014**	**0.077**	**0.025**

C

Experimental IC_{80} titres in nM

Pseudovirus	single scFv					2 scFv combinations										3 scFv combinations									
	CAP256.25	PGT121	3BNC117	10E8v4	8ANC195	CAP256.25 +PGT121	CAP256.25 +3BNC117	CAP256.25 +10E8v4	CAP256.25 +8ANC195	PGT121 +3BNC117	PGT121 +10E8v4	PGT121 +8ANC195	3BNC117 +8ANC195	3BNC117 +10E8v4	10E8v4 +8ANC195	CAP256.25 +PGT121 +10E8v4	CAP256.25 3BNC117 +10E8v4	CAP256.25 +10E8v4 +8ANC195	PGT121 +3BNC117 +10E8v4	PGT121 +10E8v4 +8ANC195	3BNC117 +10E8v4 +8ANC195	CAP256.25 +3BNC117 +PGT121	CAP256.25 +PGT121 8ANC195	CAP256.25 +3BNC117 +8ANC195	3BNC117 +PGT121 +8ANC195
Q23.17	350	0.50	5.1	53	593	0.66	4.9	12	245	0.60	0.84	0.75	5.5	3.7	48	0.31	2.5	15	0.33	0.35	3.8	0.29	0.31	2.7	0.34
Q168.A2	>1000	>1000	3.4	52	90	>1000	2.7	17	51	3.1	60	112	2.9	2.7	20	23	1.6	9.5	2.3	15	2.6	2.7	34	2.0	2.9
Q842.D12	>1000	13	1.1	69	>1000	8.9	0.90	56	566	0.54	3.8	7.4	0.85	0.71	69	6.7	0.76	38	0.56	4.8	0.85	0.66	9.1	0.76	0.60
AC10.0.29	>1000	8.2	>1000	13	>1000	5.6	>1000	10	349	6.4	3.3	8.1	1000	12	10	2.7	9.6	7.9	2.5	2.3	10	6.2	7.1	282	5.1
RHPA4259.7	>1000	2.4	2.1	40	36	4.6	1.8	30	35	1.2	2.8	2.8	2.3	2.2	14	2.0	1.2	11	0.66	1.1	1.4	0.61	1.4	1.5	0.81
PVO.4	325	24	4.7	87	326	20	3.9	29	48	3.1	13	15	4.8	4.1	32	12	2.9	16	2.8	8.3	3.7	2.9	16	4.2	3.6
6535.3	>1000	0.41	327	19	104	0.40	1000	13	59	0.38	0.37	0.53	63	14	8.0	0.26	15	13	0.48	0.26	9.1	0.29	0.27	64	0.48
Du156.12	4.6	1.1	281	6.6	347	0.64	3.8	1.4	5.4	1.3	0.79	1.5	77	6.5	6.3	0.44	1.7	1.8	0.68	0.45	10	0.41	0.37	7.4	0.87
ZM53M.PB12	0.43	149	379	69	28	0.53	0.56	0.53	0.52	28	5.7	41	20	32	31	0.29	0.35	0.30	7.0	3.9	12	0.24	0.20	0.41	9.8
ZM233M.PB6	23	>1000	490	15	879	28	18	2.7	21	340	13	>1000	250	26	14	4.3	4.4	3.0	17	14	14	48	77	29	95
ZM249M.PL1	>1000	732	16	80	1000	196	9.9	21	348	26	25	172	14	10.0	64	21	5.4	20	9.0	35	12	12	92	16	27
CAP8.6F	>1000	2.0	>1000	22	364	1.2	>1000	12	302	1.6	0.95	1.3	423	33	16	0.81	19	9.2	1.3	0.63	17	1.4	1.0	260	2.0
CAP61.4.22.F10A	1000	3.6	252	60	>1000	1.8	158	22	399	2.4	1.3	1.8	291	39	40	1.2	24	18	1.6	0.91	38	2.1	1.5	175	2.1
CAP84.32	>1000	0.41	>1000	35	>1000	0.53	>1000	22	>1000	0.48	0.35	0.50	995	40	47	0.39	29	28	0.34	0.38	21	0.33	0.30	475	1.1
CAP88.B5	>1000	>1000	>1000	1.4	482	>1000	>1000	0.88	990	433	0.59	328	629	0.81	1.2	0.85	0.44	0.41	0.91	0.57	0.67	440	104	426	228
CAP256.SU	0.45	2.1	57	87	32	0.34	0.51	0.46	0.47	2.0	1.6	2.2	25	22	30	0.26	0.39	0.35	1.6	1.3	9.9	0.40	0.19	0.46	2.1
ConC	1.6	1.6	233	19	253	0.44	3.2	1.5	3.6	0.92	0.60	1.1	114	10	9.3	0.30	0.94	0.91	0.78	0.77	7.4	0.31	0.27	2.6	0.92
**Geometric mean**	**16**	**4.5**	**36**	**30**	**199**	**2.4**	**5.8**	**7.0**	**49**	**3.7**	**2.4**	**5.6**	**45**	**8.4**	**19**	**1.4**	**2.8**	**5.3**	**1.4**	**1.8**	**6.4**	**1.6**	**2.5**	**13**	**3.1**

D

Experimental IC_{50} titres in µg/mL

Pseudovirus	single scFv: CAP256.25	PGT121	3BNC117	10E8v4	8ANC195
Q23.17	11	0.014	0.15	1.5	17
Q168.A2	>30	>30	0.097	1.5	2.6
Q842.D12	>30	0.38	0.032	2.0	>30
AC10.0.29	>30	0.24	>30	0.38	>30
RHPA4259.7	>30	0.068	0.060	1.2	1.0
PVO.4	10	0.70	0.13	2.6	9.5
6535.3	>30	0.012	9.3	0.56	3.0
Du156.12	0.14	0.032	8.0	0.19	10
ZM53M.PB12	0.013	4.3	11	2.0	0.81
ZM233M.PB6	0.71	>30	14	0.45	25
ZM249M.PL1	>30	21	0.45	2.4	29
CAP8.6F	>30	0.058	>30	0.65	11
CAP61.4.22.F10A	31	0.10	7.2	1.8	>30
CAP84.32	>30	0.012	>30	1.0	>30
CAP88.B5	>30	>30	>30	0.040	14
CAP256.SU	0.014	0.061	1.6	2.5	0.91
ConC	0.050	0.047	6.6	0.56	7.3
**Geometric mean**	**0.49**	**0.13**	**1.0**	**0.86**	**5.8**

Pseudovirus	2 scFv combinations: CAP256.25 +PGT121	CAP256.25 +3BNC117	CAP256.25 +10E8v4	CAP256.25 +8ANC195	PGT121 +3BNC117	PGT121 +10E8v4	PGT121 +8ANC195	3BNC117 +8ANC195	3BNC117 +10E8v4	10E8v4 +8ANC195
Q23.17	0.020	0.15	0.36	7.4	0.017	0.025	0.022	0.16	0.11	1.4
Q168.A2	>30	0.082	0.53	1.6	0.089	1.7	3.3	0.084	0.079	0.59
Q842.D12	0.27	0.027	1.7	17	0.016	0.11	0.21	0.025	0.020	2.0
AC10.0.29	0.17	>30	0.31	11	0.18	0.096	0.24	29	0.36	0.30
RHPA4259.7	0.14	0.053	0.92	1.0	0.035	0.081	0.082	0.066	0.064	0.41
PVO.4	0.61	0.12	0.88	1.4	0.088	0.37	0.42	0.14	0.12	0.93
6535.3	0.012	30	0.39	1.8	0.011	0.011	0.015	1.8	0.40	0.23
Du156.12	0.019	0.11	0.044	0.16	0.036	0.023	0.042	2.2	0.19	0.18
ZM53M.PB12	0.016	0.017	0.016	0.016	0.79	0.17	1.2	0.58	0.94	0.90
ZM233M.PB6	0.86	0.54	0.083	0.64	9.7	0.38	>30	7.2	0.74	0.42
ZM249M.PL1	5.9	0.30	0.65	10	0.75	0.73	5.0	0.41	0.29	1.9
CAP8.6F	0.036	>30	0.37	9.1	0.046	0.028	0.037	12	0.95	0.47
CAP61.4.22.F10A	0.053	4.7	0.65	12	0.069	0.038	0.052	8.4	1.1	1.2
CAP84.32	0.016	>30	0.65	>30	0.014	0.010	0.014	29	1.2	1.4
CAP88.B5	>30	>30	0.027	30	12	0.017	9.5	18	0.023	0.035
CAP256.SU	0.010	0.015	0.014	0.014	0.057	0.047	0.064	0.73	0.63	0.87
ConC	0.013	0.097	0.044	0.11	0.026	0.018	0.031	3.3	0.29	0.27
**Geometric mean**	**0.071**	**0.17**	**0.21**	**1.5**	**0.11**	**0.070**	**0.16**	**1.3**	**0.24**	**0.55**

Pseudovirus	3 scFv combinations: CAP256.25 +PGT121 +10E8v4	CAP256.25 3BNC117 +10E8v4	CAP256.25 +10E8v4 +8ANC195	PGT121 +3BNC117 +10E8v4	PGT121 +10E8v4 +8ANC195	3BNC117 +10E8v4 +8ANC195	CAP256.25 +3BNC117 +PGT121	CAP256.25 +PGT121 8ANC195	CAP256.25 +3BNC117 +8ANC195	3BNC117 +PGT121 +8ANC195
Q23.17	0.009	0.075	0.45	0.010	0.010	0.11	0.008	0.009	0.079	0.010
Q168.A2	0.70	0.049	0.28	0.065	0.44	0.074	0.081	1.0	0.058	0.082
Q842.D12	0.20	0.022	1.1	0.016	0.14	0.025	0.019	0.27	0.022	0.017
AC10.0.29	0.080	0.29	0.24	0.073	0.068	0.30	0.18	0.21	8.3	0.15
RHPA4259.7	0.059	0.034	0.32	0.019	0.032	0.040	0.018	0.041	0.045	0.023
PVO.4	0.36	0.085	0.49	0.080	0.24	0.11	0.085	0.47	0.13	0.10
6535.3	0.008	0.45	0.38	0.014	0.008	0.26	0.009	0.008	1.9	0.014
Du156.12	0.013	0.052	0.055	0.020	0.013	0.29	0.012	0.011	0.22	0.025
ZM53M.PB12	0.009	0.010	0.009	0.20	0.11	0.34	0.007	0.006	0.012	0.28
ZM233M.PB6	0.13	0.13	0.090	0.50	0.41	0.41	1.4	2.3	0.85	2.7
ZM249M.PL1	0.62	0.16	0.60	0.26	1.0	0.36	0.35	2.7	0.46	0.76
CAP8.6F	0.024	0.56	0.27	0.037	0.018	0.48	0.042	0.030	7.7	0.057
CAP61.4.22.F10A	0.037	0.72	0.53	0.047	0.026	1.1	0.061	0.046	5.2	0.061
CAP84.32	0.012	0.87	0.83	0.010	0.011	0.60	0.010	0.009	14	0.032
CAP88.B5	0.025	0.013	0.012	0.026	0.017	0.019	13	3.1	13	6.6
CAP256.SU	0.008	0.011	0.010	0.045	0.037	0.29	0.012	0.006	0.013	0.060
ConC	0.009	0.028	0.027	0.022	0.022	0.21	0.009	0.008	0.077	0.027
**Geometric mean**	**0.041**	**0.084**	**0.16**	**0.042**	**0.051**	**0.19**	**0.049**	**0.074**	**0.38**	**0.088**

Supplementary 3: Breadth and Potency of single scFv against a 45 virus panel

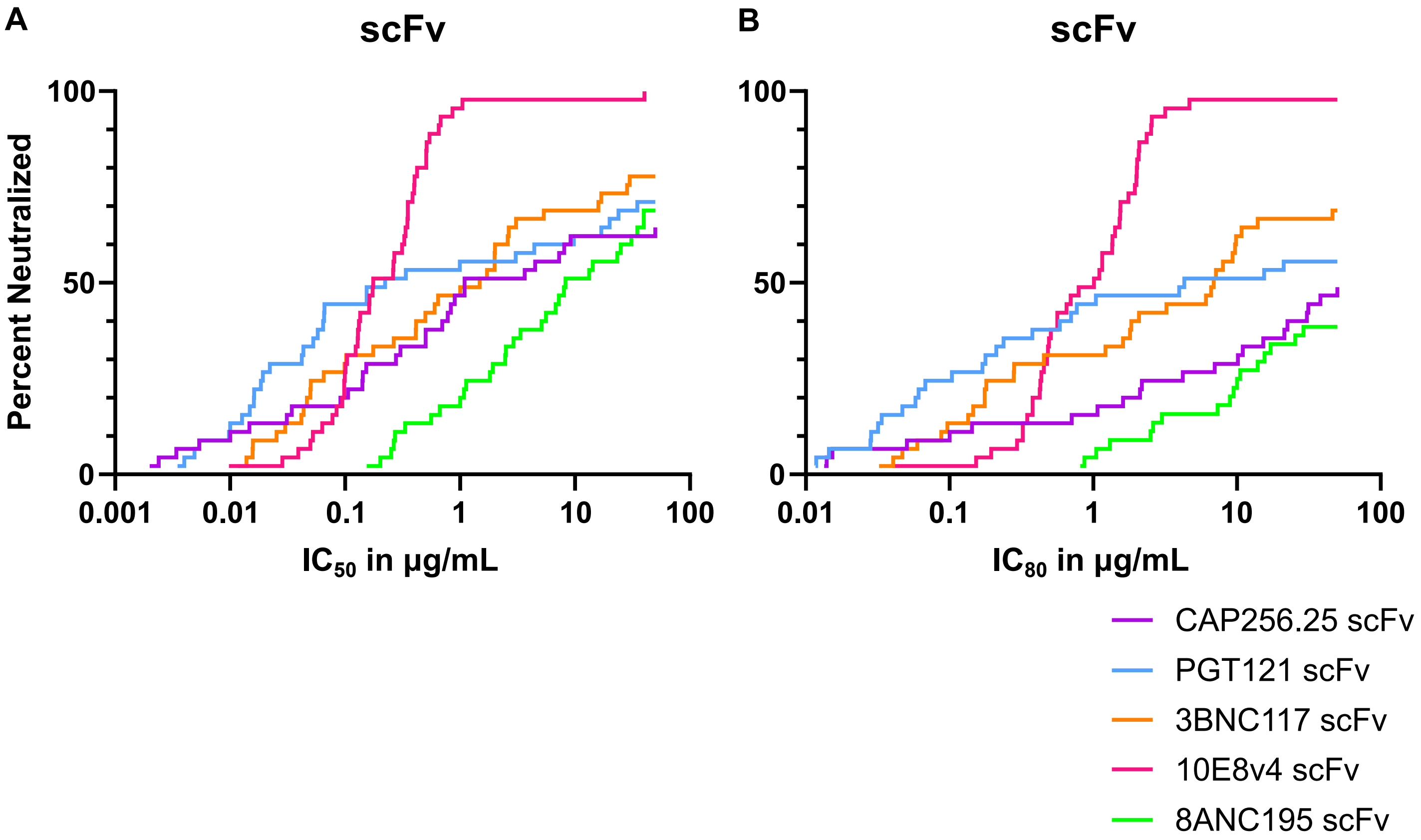

Supplementary Figure 4

A

Combination IC_{50} titres in nanomolar (nM) (Bliss-Hill model)

Pseudovirus	2 scFv combinations										3 scFv combinations									
	3BNC117 +8ANC195	3BNC117 +10E8v4	CAP256.25 +3BNC117	3BNC117 +PGT121	10E8v4 +8ANC195	CAP256.25 +8ANC195	PGT121 +8ANC195	CAP256.25 +10E8v4	PGT121 +10E8v4	CAP256.25 +PGT121	3BNC117 +10E8v4 +8ANC195	CAP256.25 +3BNC117 +8ANC195	3BNC117 +PGT121 +8ANC195	CAP256.25 +3BNC117 +10E8v4	3BNC117 +PGT121 +10E8v4	CAP256.25 +3BNC117 +PGT121	CAP256.25 +10E8v4 +8ANC195	PGT121 +10E8v4 +8ANC195	CAP256.25 +PGT121 +8ANC195	CAP256.25 +PGT121 +10E8v4
Q23.17	1.47	1.28	0.73	0.17	14.17	9.40	0.19	3.28	0.19	0.14	1.28	0.73	0.17	0.67	0.17	0.13	3.27	0.19	0.14	0.14
Q168.A2	0.99	1.01	0.70	1.06	9.04	6.77	22.95	6.57	17.33	34.93	0.95	0.66	0.99	0.68	1.01	0.70	4.14	9.04	6.77	6.57
Q259.D2.17	>1595	35.71	22.33	34.60	35.71	22.33	34.60	8.67	10.39	2.44	35.71	22.33	34.60	8.67	10.39	2.44	8.67	10.39	2.44	2.12
Q461.E2	1.70	1.49	1.38	1.76	14.24	19.81	276.24	7.33	17.33	28.71	1.45	1.35	1.70	1.21	1.49	1.38	6.58	14.24	19.81	7.33
Q769.D22	0.42	0.37	0.49	0.49	2.08	11.50	11.50	5.53	5.53	>1595	0.33	0.42	0.42	0.37	0.37	0.49	2.08	2.08	11.50	5.53
Q842.D12	0.41	0.40	0.39	0.29	17.96	87.39	1.99	11.41	1.63	1.61	0.40	0.39	0.29	0.38	0.28	0.28	11.16	1.62	1.61	1.37
BG505.W6M 332N	0.49	0.43	0.36	0.32	1.91	1.76	0.51	1.09	0.47	0.41	0.39	0.32	0.30	0.30	0.28	0.24	0.81	0.44	0.38	0.35
CAAN5342.A2	22.68	11.04	22.68	1.97	29.34	>1595	2.29	29.34	1.59	2.29	11.04	22.68	1.97	11.04	1.46	1.97	29.34	1.59	2.29	1.59
AC10.0.29	62.68	3.45	233.03	1.46	2.18	18.16	0.96	2.86	0.83	1.25	2.18	18.16	0.96	2.86	0.83	1.25	1.85	0.62	0.84	0.75
RHPA4259.7	0.50	0.53	0.56	0.19	4.43	8.66	0.39	11.99	0.42	0.44	0.48	0.50	0.19	0.53	0.19	0.19	4.43	0.38	0.39	0.42
TRO.11	1.15	1.22	1.63	0.85	3.13	6.98	1.49	8.93	1.60	2.23	0.94	1.15	0.71	1.22	0.74	0.85	3.13	1.18	1.49	1.60
PVO.4	1.44	1.39	1.15	1.05	13.48	11.76	4.35	7.57	3.73	2.80	1.32	1.09	1.01	1.06	0.99	0.83	5.73	3.25	2.46	2.25
SC422661.8	2.82	1.03	3.48	3.22	1.34	115.76	29.99	1.71	1.59	105.45	0.89	2.82	2.63	1.03	0.99	3.22	1.34	1.27	29.99	1.59
REJO.67	5.21	2.51	9.33	9.33	3.17	39.09	39.09	4.51	4.51	>1595	1.98	5.21	5.21	2.51	2.51	9.33	3.17	3.17	39.09	4.51
TRJO4551.18	69.06	10.84	91.48	75.37	12.56	491.44	251.17	13.19	12.76	830.45	10.43	69.06	59.88	10.84	10.56	75.37	12.56	12.17	251.17	12.76
WITO.33	0.89	0.68	0.89	0.89	4.43	>1595	>1595	4.43	4.43	>1595	0.68	0.89	0.89	0.68	0.68	0.89	4.43	4.43	>1595	4.43
THRO4156.18	70.32	4.04	70.32	70.32	4.23	>1595	>1595	4.23	4.23	>1595	4.04	70.32	70.32	4.04	4.04	70.32	4.23	4.23	>1595	4.23
QH0692.42	3.35	2.59	3.59	3.59	9.78	233.94	233.94	11.99	11.99	>1595	2.46	3.35	3.35	2.59	2.59	3.59	9.78	9.78	233.94	11.99
6535.3	19.20	5.56	188.03	0.12	3.55	19.35	0.12	5.56	0.12	0.12	3.55	19.20	0.12	5.56	0.12	0.12	3.55	0.11	0.12	0.12
Du151.2	1053.74	8.92	1.10	0.17	8.99	1.10	0.17	0.83	0.17	0.07	8.92	1.10	0.17	0.83	0.17	0.07	0.83	0.17	0.07	0.07
Du156.12	25.01	1.21	0.44	0.50	1.29	0.46	0.51	0.23	0.36	0.22	1.17	0.43	0.49	0.22	0.35	0.22	0.23	0.35	0.22	0.16
Du172.17	1369.86	1.79	>1595	337.88	1.78	1369.86	228.84	1.79	1.74	337.88	1.78	1369.86	228.84	1.79	1.74	337.88	1.78	1.73	228.84	1.74
Du422.1	>1595	4.39	8.85	7.72	4.39	8.85	7.72	1.52	1.74	0.96	4.39	8.85	7.72	1.52	1.74	0.96	1.52	1.74	0.96	0.62
ZM53M.PB12	3.91	6.72	0.06	1.56	2.44	0.06	0.72	0.06	0.92	0.03	2.06	0.06	0.64	0.06	0.79	0.03	0.05	0.50	0.03	0.03
ZM109F.PB4	>1595	3.36	35.09	>1595	3.36	35.09	>1595	1.37	3.36	35.09	3.36	35.09	>1595	1.37	3.36	35.09	1.37	3.36	35.09	1.37
ZM135M.PL10A	1.77	0.79	1.77	1.77	2.16	>1595	>1595	2.16	2.16	>1595	0.79	1.77	1.77	0.79	0.79	1.77	2.16	2.16	>1595	2.16
ZM197M.PB7	17.07	0.88	0.31	17.51	0.97	0.32	1369.86	0.15	0.97	0.32	0.88	0.31	17.07	0.15	0.88	0.31	0.15	0.97	0.32	0.15
ZM214M.PL15	1.75	1.12	1.35	0.56	1.46	2.04	0.62	1.14	0.51	0.51	0.56	0.56	0.31	0.47	0.29	0.27	0.50	0.29	0.27	0.25
ZM233M.PB6	29.57	3.13	0.94	78.60	2.58	0.75	58.68	0.47	3.35	0.98	2.42	0.72	26.18	0.46	3.08	0.93	0.41	2.55	0.75	0.47
ZM249M.PL1	2.27	1.42	0.72	0.72	12.62	12.96	10.08	2.57	2.42	0.85	1.41	0.72	0.72	0.55	0.55	0.26	2.52	2.38	0.85	0.55
CAP8.6F	100.72	6.02	257.00	0.34	5.54	45.76	0.34	4.78	0.31	0.32	5.54	45.76	0.34	4.78	0.31	0.32	4.47	0.30	0.31	0.29
CAP45.2.00.G3	804.70	3.56	3.39	1211.07	3.53	3.28	391.83	0.82	3.54	3.30	3.53	3.28	391.83	0.82	3.54	3.30	0.82	3.51	3.19	0.82
CAP61.4.22.F10A	73.30	9.64	46.92	0.67	10.10	84.60	0.67	7.89	0.54	0.64	9.18	40.26	0.67	7.37	0.54	0.64	7.57	0.54	0.64	0.52
CAP63.A9	21.07	7.22	2.27	3.22	17.54	4.90	5.32	2.47	3.37	1.23	7.22	2.27	3.22	1.63	2.40	0.97	2.47	3.37	1.23	1.03
CAP84.32	562.00	11.37	149.21	0.14	11.48	293.00	0.14	8.91	0.14	0.14	11.37	149.21	0.14	8.85	0.14	0.14	8.91	0.14	0.14	0.14
CAP85.9	347.48	2.87	597.12	1.83	2.87	1207.05	1.83	2.89	0.89	1.83	2.85	347.48	1.82	2.87	0.89	1.83	2.87	0.89	1.83	0.89
CAP88.B5	148.87	0.34	998.88	113.85	0.34	176.88	64.82	0.34	0.32	153.33	0.34	148.87	59.46	0.34	0.32	113.85	0.34	0.32	64.82	0.32
CAP206.8	>1595	2.65	4.51	692.04	2.65	4.51	692.04	0.96	2.62	4.37	2.65	4.51	692.04	0.96	2.62	4.37	0.96	2.62	4.37	0.96
CAP210.2.00.E8	14.45	2.26	0.07	14.45	11.93	0.11	>1595	0.11	11.93	0.11	2.26	0.07	14.45	0.07	2.26	0.07	0.11	11.93	0.11	0.11
CAP228.51	>1595	1370.00	24.93	>1595	1370.00	24.93	>1595	22.30	1370.00	24.93	1370.00	24.93	>1595	22.30	1370.00	24.93	22.30	1370.00	24.93	22.30
CAP239.2.00.G3J	>1595	3.23	2.91	0.77	3.23	2.91	0.77	0.86	0.44	0.32	3.23	2.91	0.77	0.86	0.44	0.32	0.86	0.44	0.32	0.24
CAP244.D3	49.21	6.96	70.25	70.25	8.90	193.70	193.70	9.19	9.19	>1595	6.82	49.21	49.21	6.96	6.96	70.25	8.90	8.90	193.70	9.19
CAP255.2.00.16J	31.52	6.66	35.12	0.33	13.12	862.18	0.35	13.62	0.33	0.35	6.54	31.52	0.33	6.66	0.31	0.33	13.12	0.33	0.35	0.33
CAP256.SU	5.05	7.68	0.08	0.60	6.06	0.08	0.60	0.08	0.62	0.07	3.93	0.08	0.56	0.08	0.58	0.07	0.08	0.58	0.07	0.07
ConC	19.78	5.45	0.17	0.56	4.18	0.16	0.52	0.16	0.51	0.11	3.95	0.16	0.52	0.16	0.51	0.11	0.15	0.48	0.11	0.11
**Geometric mean**	**12.37**	**2.93**	**4.40**	**2.75**	**5.51**	**11.95**	**5.55**	**2.33**	**1.77**	**1.49**	**2.59**	**3.75**	**2.31**	**1.16**	**1.04**	**1.13**	**1.98**	**1.57**	**1.78**	**0.81**
**Breadth**	**87%**	**100%**	**98%**	**96%**	**100%**	**91%**	**87%**	**100%**	**100%**	**84%**	**100%**	**100%**	**96%**	**100%**	**100%**	**100%**	**100%**	**100%**	**93%**	**100%**

B

Combination IC_{50} titres in µg/mL (Bliss-Hill model)

	**2 scFv combinations**										**3 scFv combinations**									
**Pseudovirus**	**3BNC117 +8ANC195**	**3BNC117 +10E8v4**	**CAP256.25 +3BNC117**	**3BNC117 +PGT121**	**10E8v4 +8ANC195**	**CAP256.25 +8ANC195**	**PGT121 +8ANC195**	**CAP256.25 +10E8v4**	**PGT121 +10E8v4**	**CAP256.25 +PGT121**	**3BNC117 +10E8v4 +8ANC195**	**CAP256.25 +3BNC117 +8ANC195**	**3BNC117 +PGT121 +8ANC195**	**CAP256.25 +3BNC117 +10E8v4**	**3BNC117 +PGT121 +10E8v4**	**CAP256.25 +3BNC117 +PGT121**	**CAP256.25 +10E8v4 +8ANC195**	**PGT121 +10E8v4 +8ANC195**	**CAP256.25 +PGT121 +8ANC195**	**CAP256.25 +PGT121 +10E8v4**
Q23.17	0.042	0.037	0.022	0.0048	0.41	0.28	0.0055	0.099	0.0054	0.0043	0.037	0.022	0.0048	0.020	0.0048	0.0038	0.098	0.0054	0.0043	0.0042
Q168.A2	0.028	0.029	0.021	0.030	0.26	0.20	0.66	0.20	0.50	1.1	0.027	0.020	0.028	0.020	0.029	0.021	0.12	0.26	0.20	0.20
Q259.D2.17	>50	1.0	0.67	0.99	1.0	0.67	1.0	0.26	0.30	0.073	1.0	0.66	1.00	0.26	0.30	0.072	0.26	0.30	0.073	0.063
Q461.E2	0.049	0.043	0.041	0.050	0.42	0.60	8.0	0.22	0.50	0.86	0.042	0.040	0.049	0.036	0.043	0.041	0.20	0.41	0.59	0.22
Q769.D22	0.012	0.011	0.015	0.014	0.061	0.35	0.33	0.17	0.16	>50	0.0094	0.012	0.012	0.011	0.011	0.014	0.062	0.060	0.34	0.17
Q842.D12	0.012	0.012	0.012	0.0082	0.52	2.6	0.058	0.35	0.047	0.049	0.012	0.012	0.0083	0.011	0.0081	0.0081	0.33	0.047	0.048	0.041
BG505.W6M 332N	0.014	0.013	0.011	0.0091	0.056	0.053	0.015	0.033	0.014	0.012	0.011	0.0095	0.0086	0.0088	0.0081	0.0072	0.024	0.013	0.011	0.010
CAAN5342.A2	0.65	0.32	0.68	0.057	0.86	>50	0.066	0.89	0.046	0.069	0.32	0.67	0.057	0.33	0.042	0.058	0.88	0.046	0.068	0.047
AC10.0.29	1.8	0.100	7.0	0.042	0.063	0.55	0.028	0.087	0.024	0.038	0.063	0.54	0.028	0.085	0.024	0.037	0.055	0.018	0.025	0.022
RHPA4259.7	0.014	0.015	0.017	0.0055	0.13	0.26	0.011	0.36	0.012	0.013	0.014	0.015	0.0053	0.016	0.0055	0.0057	0.13	0.011	0.012	0.013
TRO.11	0.033	0.035	0.049	0.024	0.091	0.21	0.043	0.27	0.047	0.067	0.027	0.034	0.020	0.036	0.021	0.025	0.093	0.034	0.044	0.048
PVO.4	0.041	0.040	0.034	0.030	0.39	0.35	0.13	0.23	0.11	0.084	0.038	0.032	0.029	0.031	0.028	0.025	0.17	0.094	0.073	0.067
SC422661.8	0.081	0.030	0.10	0.092	0.039	3.5	0.87	0.052	0.046	3.2	0.026	0.083	0.076	0.031	0.029	0.095	0.040	0.037	0.89	0.048
REJO.67	0.15	0.073	0.28	0.27	0.092	1.2	1.1	0.14	0.13	>50	0.057	0.15	0.15	0.075	0.073	0.28	0.095	0.092	1.2	0.13
TRJO4551.18	2.0	0.31	2.7	2.2	0.37	15	7.3	0.40	0.37	25	0.30	2.0	1.7	0.32	0.31	2.2	0.38	0.35	7.5	0.38
WITO.33	0.026	0.020	0.027	0.026	0.13	>50	>50	0.13	0.13	>50	0.020	0.026	0.026	0.020	0.020	0.026	0.13	0.13	>50	0.13
THRO4156.18	2.0	0.12	2.1	2.0	0.12	>50	>50	0.13	0.12	>50	0.12	2.1	2.0	0.12	0.12	2.1	0.13	0.12	>50	0.13
QH0692.42	0.096	0.075	0.11	0.10	0.28	7.1	6.8	0.36	0.35	>50	0.071	0.099	0.097	0.077	0.075	0.11	0.29	0.28	7.0	0.36
6535.3	0.55	0.16	5.6	0.0034	0.10	0.58	0.0034	0.17	0.0034	0.0036	0.10	0.57	0.0034	0.17	0.0034	0.0035	0.11	0.0033	0.0035	0.0035
Du151.2	30	0.26	0.033	0.0049	0.26	0.033	0.0049	0.025	0.0049	0.0020	0.26	0.032	0.0049	0.025	0.0048	0.0019	0.025	0.0049	0.0019	0.0019
Du156.12	0.72	0.035	0.013	0.014	0.038	0.014	0.015	0.0069	0.010	0.0067	0.034	0.013	0.014	0.0066	0.010	0.0064	0.0068	0.010	0.0066	0.0049
Du172.17	39	0.052	>50	9.7	0.052	41	6.6	0.054	0.051	10	0.052	41	6.6	0.053	0.050	10.0	0.053	0.050	6.8	0.052
Du422.1	>50	0.13	0.26	0.22	0.13	0.27	0.22	0.046	0.051	0.029	0.13	0.26	0.22	0.045	0.050	0.028	0.045	0.051	0.029	0.018
ZM53M.PB12	0.11	0.19	0.0018	0.045	0.071	0.0017	0.021	0.0017	0.027	0.0009	0.060	0.0016	0.018	0.0017	0.023	0.0009	0.0016	0.015	0.0009	0.0009
ZM109F.PB4	>50	0.097	1.0	>50	0.098	1.1	>50	0.042	0.098	1.1	0.097	1.0	>50	0.041	0.097	1.0	0.041	0.098	1.0	0.041
ZM135M.PL10A	0.051	0.023	0.053	0.051	0.063	>50	>50	0.066	0.063	>50	0.023	0.052	0.051	0.023	0.023	0.052	0.065	0.063	>50	0.064
ZM197M.PB7	0.49	0.025	0.0092	0.50	0.028	0.0097	40	0.0046	0.028	0.0096	0.025	0.0090	0.49	0.0045	0.025	0.0090	0.0046	0.028	0.0095	0.0046
ZM214M.PL15	0.050	0.032	0.040	0.016	0.042	0.062	0.018	0.035	0.015	0.015	0.016	0.017	0.0089	0.014	0.0082	0.0079	0.015	0.0083	0.0079	0.0074
ZM233M.PB6	0.85	0.090	0.028	2.3	0.075	0.023	1.7	0.014	0.097	0.030	0.070	0.021	0.75	0.014	0.089	0.027	0.012	0.074	0.022	0.014
ZM249M.PL1	0.065	0.041	0.022	0.021	0.37	0.39	0.29	0.078	0.070	0.026	0.041	0.021	0.021	0.016	0.016	0.0078	0.075	0.069	0.025	0.016
CAP8.6F	2.9	0.17	7.7	0.0098	0.16	1.4	0.0098	0.14	0.0089	0.0095	0.16	1.4	0.0097	0.14	0.0088	0.0093	0.13	0.0088	0.0093	0.0085
CAP45.2.00.G3	23	0.10	0.10	35	0.10	0.099	11	0.025	0.10	0.099	0.10	0.097	11	0.024	0.10	0.098	0.024	0.10	0.095	0.024
CAP61.4.22.F10A	2.1	0.28	1.4	0.019	0.29	2.6	0.019	0.24	0.016	0.019	0.27	1.2	0.019	0.22	0.016	0.019	0.23	0.016	0.019	0.015
CAP63.A9	0.61	0.21	0.068	0.092	0.51	0.15	0.15	0.075	0.098	0.037	0.21	0.067	0.093	0.048	0.069	0.029	0.074	0.098	0.036	0.031
CAP84.32	16	0.33	4.5	0.0040	0.33	8.8	0.0041	0.27	0.0040	0.0041	0.33	4.4	0.0040	0.26	0.0040	0.0040	0.27	0.0040	0.0040	0.0041
CAP85.9	10.0	0.083	18	0.052	0.084	36	0.053	0.088	0.026	0.055	0.083	10	0.052	0.085	0.026	0.054	0.086	0.026	0.054	0.027
CAP88.B5	4.3	0.0098	30	3.3	0.0099	5.3	1.9	0.010	0.0094	4.6	0.0098	4.4	1.7	0.010	0.0093	3.4	0.010	0.0094	1.9	0.0096
CAP206.8	>50	0.077	0.13	20	0.077	0.14	20	0.029	0.076	0.13	0.077	0.13	20	0.029	0.076	0.13	0.029	0.076	0.13	0.029
CAP210.2.00.E8	0.42	0.065	0.0021	0.41	0.35	0.0033	>50	0.0032	0.35	0.0033	0.065	0.0021	0.42	0.0020	0.065	0.0021	0.0031	0.35	0.0033	0.0031
CAP228.51	>50	40	0.75	>50	40	0.75	>50	0.68	40	0.75	40	0.74	>50	0.66	40	0.74	0.67	40	0.74	0.67
CAP239.2.00.G3J	>50	0.093	0.087	0.022	0.094	0.088	0.022	0.026	0.013	0.0097	0.093	0.086	0.022	0.026	0.013	0.0095	0.026	0.013	0.0095	0.0071
CAP244.D3	1.4	0.20	2.1	2.0	0.26	5.8	5.6	0.28	0.27	>50	0.20	1.5	1.4	0.21	0.20	2.1	0.27	0.26	5.8	0.27
CAP255.2.00.16J	0.91	0.19	1.1	0.0095	0.38	26	0.010	0.41	0.0096	0.011	0.19	0.93	0.0096	0.20	0.0091	0.0098	0.39	0.0096	0.010	0.0098
CAP256.SU	0.15	0.22	0.0024	0.017	0.18	0.0024	0.017	0.0024	0.018	0.0020	0.11	0.0023	0.016	0.0023	0.017	0.0020	0.0024	0.017	0.0020	0.0020
ConC	0.57	0.16	0.0051	0.016	0.12	0.0048	0.015	0.0049	0.015	0.0034	0.11	0.0047	0.015	0.0048	0.015	0.0033	0.0046	0.014	0.0032	0.0033
**Geometric mean**	**0.36**	**0.08**	**0.13**	**0.08**	**0.16**	**0.36**	**0.16**	**0.07**	**0.05**	**0.04**	**0.07**	**0.11**	**0.07**	**0.03**	**0.03**	**0.03**	**0.06**	**0.05**	**0.05**	**0.02**
**Breadth**	**87%**	**100%**	**98%**	**96%**	**100%**	**91%**	**87%**	**100%**	**100%**	**84%**	**100%**	**100%**	**96%**	**100%**	**100%**	**100%**	**100%**	**100%**	**93%**	**100%**

C

Combination IC$_{80}$ titres in nanomolar (nM) (Bliss-Hill model)

	2 scFv combinations									
**Pseudovirus**	**3BNC117 +8ANC195**	**3BNC117 +10E8v4**	**CAP256.25 +3BNC117**	**3BNC117 +PGT121**	**10E8v4 +8ANC195**	**CAP256.25 +8ANC195**	**PGT121 +8ANC195**	**CAP256.25 +10E8v4**	**PGT121 +10E8v4**	**CAP256.25 +PGT121**
Q23.17	5.1	4.0	2.8	0.4	48.7	128.7	0.5	17.4	0.5	0.4
Q168.A2	3.0	3.0	2.4	3.4	23.5	34.4	89.9	25.7	52.1	1595.0
Q259.D2.17	>1595	108.4	1000.0	1595.0	108.4	1000.0	1595.0	41.8	46.0	49.6
Q461.E2	6.0	4.7	4.7	6.3	52.2	105.5	1595.0	27.1	71.4	223.8
Q769.D22	1.2	1.1	1.4	1.4	10.8	86.5	86.5	46.5	46.5	>1595
Q842.D12	1.1	1.1	1.1	0.8	63.9	506.0	12.8	40.2	7.4	9.2
BG505.W6M 332N	1.4	1.2	1.1	0.6	6.5	9.5	1.1	5.0	1.0	0.9
CAAN5342.A2	63.5	30.2	63.5	13.0	160.0	>1595	36.2	160.0	12.5	36.2
AC10.0.29	1595.0	13.0	>1595	8.2	8.3	193.8	5.1	10.4	2.9	6.6
RHPA4259.7	1.7	1.8	2.1	0.6	12.6	36.1	1.8	39.6	2.0	2.4
TRO.11	3.6	3.9	6.2	2.3	9.8	29.9	4.2	37.8	4.5	7.3
PVO.4	4.3	4.0	3.5	2.9	43.2	61.2	16.7	30.9	12.6	11.7
SC422661.8	8.0	3.2	9.9	8.8	7.2	1595.0	148.2	10.1	8.4	536.4
REJO.67	20.4	7.6	42.8	42.8	10.7	308.9	308.9	16.5	16.5	>1595
TRJO4551.18	216.6	32.6	346.1	246.5	41.9	2014.4	780.1	46.2	43.2	>1595
WITO.33	3.1	2.0	3.1	3.1	17.3	>1595	>1595	17.3	17.3	>1595
THRO4156.18	214.3	11.7	214.3	214.3	12.9	>1595	>1595	12.9	12.9	>1595
QH0692.42	9.1	6.5	9.9	9.9	35.9	1595.0	1595.0	48.5	48.5	>1595
6535.3	85.1	19.1	327.0	0.4	10.8	103.8	0.4	19.2	0.4	0.4
Du151.2	>1595	26.6	46.4	1.0	27.0	51.5	1.0	6.8	0.9	0.4
Du156.12	85.7	5.4	3.7	1.1	6.0	4.2	1.1	1.1	0.8	0.6
Du172.17	>1595	14.5	>1595	1595.0	14.2	>1595	796.4	14.5	13.1	1595.0
Du422.1	>1595	14.6	487.8	137.4	14.6	487.8	137.4	6.7	6.6	13.6
ZM53M.PB12	16.9	30.3	0.4	26.5	9.6	0.4	6.0	0.4	9.8	0.2
ZM109F.PB4	>1595	23.8	1595.0	>1595	23.8	1595.0	>1595	9.5	23.8	1595.0
ZM135M.PL10A	6.2	2.4	6.2	6.2	11.1	>1595	>1595	11.1	11.1	>1595
ZM197M.PB7	61.4	3.9	2.5	64.8	5.2	3.2	>1595	0.8	5.2	3.2
ZM214M.PL15	17.7	7.8	13.8	4.3	14.2	41.6	6.7	11.3	3.8	5.5
ZM233M.PB6	130.3	12.9	14.7	276.5	10.7	10.7	321.7	3.0	14.8	20.2
ZM249M.PL1	15.0	6.8	5.6	5.3	61.1	185.2	150.3	21.0	19.0	26.4
CAP8.6F	364.3	22.3	>1595	2.0	18.6	170.9	2.0	17.2	1.4	1.8
CAP45.2.00.G3	>1595	11.9	683.7	>1595	11.7	211.0	1213.6	4.7	11.7	253.3
CAP61.4.22.F10A	182.6	39.3	130.0	3.5	50.6	340.3	3.6	36.1	2.4	3.3
CAP63.A9	113.8	24.0	17.4	12.2	70.8	134.0	26.6	15.8	11.8	7.2
CAP84.32	>1595	33.7	583.3	0.4	34.6	>1595	0.4	26.8	0.4	0.4
CAP85.9	984.2	15.9	>1595	6.1	16.0	>1595	6.1	16.3	2.7	6.2
CAP88.B5	369.0	1.4	>1595	589.1	1.4	481.9	210.0	1.4	1.3	1595.0
CAP206.8	>1595	11.1	69.5	>1595	11.1	69.5	>1595	4.2	10.8	56.0
CAP210.2.00.E8	>1595	18.4	0.3	1595.0	68.1	0.5	>1595	0.4	68.1	0.5
CAP228.51	>1595	>1595	1595.0	>1595	>1595	1595.0	>1595	477.4	>1595	1595.0
CAP239.2.00.G3J	>1595	16.8	34.2	5.9	16.8	34.2	5.9	4.2	2.1	1.8
CAP244.D3	149.3	24.8	340.9	340.9	34.8	536.5	536.5	39.4	39.4	>1595
CAP255.2.00.16J	173.4	26.1	242.6	0.9	60.1	>1595	1.0	66.5	0.9	1.0
CAP256.SU	13.9	22.3	0.4	1.9	16.6	0.4	1.8	0.4	2.0	0.3
ConC	68.4	16.1	1.6	1.6	12.6	1.4	1.5	1.2	1.4	0.5
**Geometric mean**	**27.6**	**9.3**	**19.3**	**9.6**	**19.6**	**75.4**	**21.3**	**11.7**	**6.4**	**9.8**
**Breadth**	**76%**	**98%**	**89%**	**91%**	**98%**	**82%**	**82%**	**100%**	**98%**	**82%**

	3 scFv combinations									
**Pseudovirus**	**3BNC117 +10E8v4 +8ANC195**	**CAP256.25 +3BNC117 +8ANC195**	**3BNC117 +PGT121 +8ANC195**	**CAP256.25 +3BNC117 +10E8v4**	**3BNC117 +PGT121 +10E8v4**	**CAP256.25 +3BNC117 +PGT121**	**CAP256.25 +10E8v4 +8ANC195**	**PGT121 +10E8v4 +8ANC195**	**CAP256.25 +PGT121 +8ANC195**	**CAP256.25 +PGT121 +10E8v4**
Q23.17	4.0	2.8	0.4	2.4	0.4	0.3	17.1	0.5	0.4	0.4
Q168.A2	2.7	2.2	3.0	2.2	3.0	2.4	14.4	23.5	34.4	25.7
Q259.D2.17	108.4	1000.0	1595.0	41.8	46.0	49.6	41.8	46.0	49.6	18.5
Q461.E2	4.5	4.5	6.0	3.7	4.7	4.7	23.4	52.2	105.5	27.1
Q769.D22	0.9	1.2	1.2	1.1	1.1	1.4	10.8	10.8	86.5	46.5
Q842.D12	1.1	1.1	0.8	1.0	0.7	0.7	38.6	7.3	9.1	6.0
BG505.W6M 332N	1.0	1.0	0.6	0.9	0.6	0.5	3.2	0.9	0.8	0.8
CAAN5342.A2	30.2	63.5	13.0	30.2	8.4	13.0	160.0	12.5	36.2	12.5
AC10.0.29	8.3	193.8	5.1	10.4	2.9	6.6	7.0	2.3	4.3	2.6
RHPA4259.7	1.5	1.7	0.6	1.8	0.6	0.6	12.6	1.6	1.8	2.0
TRO.11	2.7	3.6	1.9	3.9	1.9	2.3	9.8	3.1	4.2	4.5
PVO.4	3.7	3.3	2.7	3.1	2.6	2.4	21.1	10.4	9.5	8.0
SC422661.8	2.8	8.0	7.3	3.2	3.1	8.8	7.2	6.3	148.2	8.4
REJO.67	5.9	20.4	20.4	7.6	7.6	42.8	10.7	10.7	308.9	16.5
TRJO4551.18	30.7	216.6	176.5	32.6	31.4	246.5	41.9	39.6	780.1	43.2
WITO.33	2.0	3.1	3.1	2.0	2.0	3.1	17.3	17.3	>1595	17.3
THRO4156.18	11.7	214.3	214.3	11.7	11.7	214.3	12.9	12.9	>1595	12.9
QH0692.42	6.1	9.1	9.1	6.5	6.5	9.9	35.9	35.9	1595.0	48.5
6535.3	10.8	85.1	0.4	19.1	0.4	0.4	10.8	0.4	0.4	0.4
Du151.2	26.6	46.4	1.0	6.8	0.9	0.4	6.8	0.9	0.4	0.4
Du156.12	5.0	3.4	1.1	1.1	0.8	0.6	1.1	0.8	0.6	0.5
Du172.17	14.2	>1595	796.4	14.5	13.1	1595.0	14.2	12.9	796.4	13.1
Du422.1	14.6	487.8	137.4	6.7	6.6	13.6	6.7	6.6	13.6	3.4
ZM53M.PB12	7.7	0.3	4.8	0.4	7.0	0.2	0.3	3.3	0.2	0.2
ZM109F.PB4	23.8	1595.0	>1595	9.5	23.8	1595.0	9.5	23.8	1595.0	9.5
ZM135M.PL10A	2.4	6.2	6.2	2.4	2.4	6.2	11.1	11.1	>1595	11.1
ZM197M.PB7	3.9	2.5	61.4	0.7	3.9	2.5	0.8	5.2	3.2	0.8
ZM214M.PL15	4.0	5.7	2.4	3.5	1.8	2.1	4.9	2.2	2.7	1.9
ZM233M.PB6	9.5	8.7	104.6	2.9	12.5	13.9	2.5	10.5	10.2	3.0
ZM249M.PL1	6.7	5.5	5.2	3.4	3.3	2.4	19.4	17.7	22.3	6.5
CAP8.6F	18.6	170.9	2.0	17.2	1.4	1.8	15.0	1.4	1.8	1.3
CAP45.2.00.G3	11.7	211.0	1213.6	4.7	11.7	253.3	4.7	11.5	150.3	4.7
CAP61.4.22.F10A	36.0	107.7	3.5	28.6	2.4	3.2	32.9	2.4	3.2	2.2
CAP63.A9	24.0	17.4	12.2	8.6	7.8	4.9	15.8	11.8	7.2	5.0
CAP84.32	33.7	583.3	0.4	26.3	0.4	0.4	26.8	0.4	0.4	0.4
CAP85.9	15.5	984.2	6.1	15.9	2.7	6.1	16.0	2.7	6.1	2.7
CAP88.B5	1.4	369.0	184.0	1.4	1.3	589.1	1.4	1.3	210.0	1.3
CAP206.8	11.1	69.5	>1595	4.2	10.8	56.0	4.2	10.8	56.0	4.1
CAP210.2.00.E8	18.4	0.3	1595.0	0.3	18.4	0.3	0.4	68.1	0.5	0.4
CAP228.51	>1595	1595.0	>1595	477.4	>1595	1595.0	477.4	>1595	1595.0	477.4
CAP239.2.00.G3J	16.8	34.2	5.9	4.2	2.1	1.8	4.2	2.1	1.8	1.1
CAP244.D3	23.3	149.3	149.3	24.8	24.8	340.9	34.8	34.8	536.5	39.4
CAP255.2.00.16J	25.2	173.4	0.9	26.1	0.8	0.9	60.1	0.9	1.0	0.9
CAP256.SU	10.1	0.4	1.6	0.4	1.7	0.3	0.4	1.7	0.3	0.3
ConC	11.3	1.3	1.5	1.2	1.4	0.5	1.1	1.3	0.4	0.4
**Geometric mean**	**8.0**	**20.6**	**8.7**	**4.9**	**3.2**	**6.1**	**9.5**	**5.4**	**11.4**	**3.8**
**Breadth**	**98%**	**98%**	**93%**	**100%**	**98%**	**100%**	**100%**	**98%**	**93%**	**100%**

D

Combination IC_{80} titres in µg/mL (Bliss-Hill model)

2 scFv combinations

Pseudovirus	3BNC117 +8ANC195	3BNC117 +10E8v4	CAP256.25 +3BNC117	3BNC117 +PGT121	10E8v4 +8ANC195	CAP256.25 +8ANC195	PGT121 +8ANC195	CAP256.25 +10E8v4	PGT121 +10E8v4	CAP256.25 +PGT121
Q23.17	0.15	0.11	0.085	0.012	1.4	3.9	0.014	0.53	0.014	0.012
Q168.A2	0.086	0.088	0.071	0.098	0.69	1.0	2.6	0.78	1.5	48
Q259.D2.17	>50	3.1	30	46	3.2	30	46	1.3	1.3	1.5
Q461.E2	0.17	0.14	0.14	0.18	1.5	3.2	46	0.82	2.1	6.7
Q769.D22	0.034	0.031	0.043	0.041	0.32	2.6	2.5	1.4	1.4	>50
Q842.D12	0.033	0.031	0.032	0.022	1.9	15	0.37	1.2	0.22	0.28
BG505.W6M 332N	0.040	0.034	0.033	0.018	0.19	0.29	0.031	0.15	0.028	0.027
CAAN5342.A2	1.8	0.87	1.9	0.37	4.7	>50	1.0	4.9	0.36	1.1
AC10.0.29	46	0.37	>50	0.24	0.24	5.8	0.15	0.32	0.084	0.20
RHPA4259.7	0.048	0.053	0.063	0.018	0.37	1.1	0.051	1.2	0.057	0.071
TRO.11	0.10	0.11	0.18	0.067	0.29	0.90	0.12	1.1	0.13	0.22
PVO.4	0.12	0.12	0.11	0.083	1.3	1.8	0.48	0.94	0.37	0.35
SC422661.8	0.23	0.093	0.29	0.25	0.21	48	4.3	0.31	0.25	16
REJO.67	0.59	0.22	1.3	1.2	0.31	9.3	8.9	0.50	0.48	>50
TRJO4551.18	6.2	0.94	10	7.1	1.2	>50	23	1.4	1.3	>50
WITO.33	0.089	0.058	0.092	0.089	0.50	>50	>50	0.52	0.50	>50
THRO4156.18	6.2	0.34	6.4	6.1	0.38	>50	>50	0.39	0.38	>50
QH0692.42	0.26	0.19	0.30	0.28	1.0	48	46	1.5	1.4	>50
6535.3	2.4	0.55	9.8	0.012	0.31	3.1	0.011	0.58	0.011	0.012
Du151.2	>50	0.77	1.4	0.028	0.79	1.6	0.029	0.21	0.026	0.012
Du156.12	2.5	0.16	0.11	0.031	0.18	0.13	0.032	0.034	0.022	0.018
Du172.17	>50	0.42	>50	46	0.41	>50	23	0.44	0.38	48
Du422.1	>50	0.42	15	3.9	0.43	15	4.0	0.20	0.19	0.41
ZM53M.PB12	0.48	0.87	0.012	0.76	0.28	0.011	0.17	0.011	0.29	0.0064
ZM109F.PB4	>50	0.69	48	>50	0.69	48	>50	0.29	0.69	48
ZM135M.PL10A	0.18	0.069	0.18	0.18	0.32	>50	>50	0.34	0.32	>50
ZM197M.PB7	1.8	0.11	0.074	1.9	0.15	0.096	>50	0.024	0.15	0.096
ZM214M.PL15	0.51	0.22	0.41	0.12	0.41	1.3	0.19	0.34	0.11	0.16
ZM233M.PB6	3.7	0.37	0.44	7.9	0.31	0.32	9.3	0.091	0.43	0.61
ZM249M.PL1	0.43	0.20	0.17	0.15	1.8	5.6	4.4	0.64	0.55	0.79
CAP8.6F	10	0.64	>50	0.057	0.54	5.2	0.057	0.52	0.042	0.054
CAP45.2.00.G3	>50	0.34	20	>50	0.34	6.4	35	0.14	0.34	7.6
CAP61.4.22.F10A	5.2	1.1	3.9	0.10	1.5	10	0.10	1.1	0.069	0.099
CAP63.A9	3.3	0.69	0.52	0.35	2.1	4.0	0.77	0.48	0.34	0.22
CAP84.32	47	0.97	17	0.012	1.0	>50	0.012	0.81	0.012	0.012
CAP85.9	28	0.46	>50	0.18	0.47	>50	0.18	0.49	0.079	0.19
CAP88.B5	11	0.040	>50	17	0.040	15	6.1	0.042	0.037	48
CAP206.8	>50	0.32	2.1	>50	0.32	2.1	>50	0.13	0.31	1.7
CAP210.2.00.E8	46	0.53	0.0098	46	2.0	0.015	>50	0.013	2.0	0.015
CAP228.51	>50	>50	48	>50	>50	48	>50	14	>50	48
CAP239.2.00.G3J	>50	0.49	1.0	0.17	0.49	1.0	0.17	0.13	0.060	0.056
CAP244.D3	4.3	0.72	10	9.8	1.0	16	16	1.2	1.1	>50
CAP255.2.00.16J	5.0	0.75	7.3	0.026	1.8	>50	0.028	2.0	0.026	0.030
CAP256.SU	0.40	0.64	0.013	0.053	0.49	0.013	0.052	0.013	0.057	0.0081
ConC	2.0	0.46	0.047	0.046	0.37	0.041	0.043	0.036	0.040	0.014
**Geometric mean**	**1.00**	**0.27**	**0.58**	**0.28**	**0.57**	**2.08**	**0.62**	**0.36**	**0.19**	**0.29**
**Breadth**	**80%**	**98%**	**89%**	**91%**	**98%**	**80%**	**82%**	**100%**	**98%**	**82%**

3 scFv combinations

Pseudovirus	3BNC117 +10E8v4 +8ANC195	CAP256.25 +3BNC117 +8ANC195	3BNC117 +PGT121 +8ANC195	CAP256.25 +3BNC117 +10E8v4	3BNC117 +PGT121 +10E8v4	CAP256.25 +3BNC117 +PGT121	CAP256.25 +10E8v4 +8ANC195	PGT121 +10E8v4 +8ANC195	CAP256.25 +PGT121 +8ANC195	CAP256.25 +PGT121 +10E8v4
Q23.17	0.11	0.084	0.012	0.072	0.012	0.0100	0.51	0.014	0.012	0.012
Q168.A2	0.079	0.064	0.086	0.066	0.088	0.071	0.43	0.68	1.0	0.77
Q259.D2.17	3.1	30	46	1.2	1.3	1.5	1.2	1.3	1.5	0.55
Q461.E2	0.13	0.13	0.17	0.11	0.14	0.14	0.70	1.5	3.1	0.81
Q769.D22	0.026	0.035	0.034	0.032	0.031	0.042	0.32	0.32	2.6	1.4
Q842.D12	0.031	0.032	0.022	0.031	0.022	0.022	1.2	0.21	0.27	0.18
BG505.W6M 332N	0.030	0.028	0.017	0.025	0.017	0.016	0.095	0.026	0.025	0.023
CAAN5342.A2	0.87	1.9	0.37	0.90	0.24	0.38	4.8	0.36	1.1	0.37
AC10.0.29	0.24	5.7	0.15	0.31	0.084	0.19	0.21	0.067	0.13	0.079
RHPA4259.7	0.044	0.050	0.017	0.055	0.018	0.019	0.38	0.045	0.052	0.059
TRO.11	0.078	0.11	0.054	0.11	0.056	0.069	0.29	0.091	0.13	0.14
PVO.4	0.11	0.097	0.079	0.092	0.076	0.070	0.63	0.30	0.28	0.24
SC422661.8	0.082	0.24	0.21	0.095	0.089	0.26	0.21	0.18	4.4	0.25
REJO.67	0.17	0.60	0.59	0.22	0.22	1.3	0.32	0.31	9.2	0.49
TRJO4551.18	0.89	6.4	5.1	0.97	0.91	7.3	1.3	1.2	23	1.3
WITO.33	0.058	0.091	0.089	0.059	0.058	0.091	0.52	0.50	>50	0.52
THRO4156.18	0.34	6.3	6.2	0.35	0.34	6.3	0.39	0.38	>50	0.39
QH0692.42	0.18	0.27	0.26	0.19	0.19	0.29	1.1	1.0	47	1.4
6535.3	0.31	2.5	0.011	0.57	0.011	0.012	0.32	0.011	0.012	0.012
Du151.2	0.77	1.4	0.028	0.20	0.026	0.012	0.20	0.026	0.012	0.012
Du156.12	0.14	0.10	0.031	0.032	0.022	0.017	0.033	0.022	0.018	0.014
Du172.17	0.41	>50	23	0.43	0.38	47	0.42	0.37	24	0.39
Du422.1	0.42	14	4.0	0.20	0.19	0.40	0.20	0.19	0.40	0.10
ZM53M.PB12	0.22	0.010	0.14	0.011	0.20	0.0062	0.0097	0.097	0.0057	0.0059
ZM109F.PB4	0.69	47	>50	0.28	0.69	47	0.28	0.69	47	0.28
ZM135M.PL10A	0.069	0.18	0.18	0.071	0.069	0.18	0.33	0.32	>50	0.33
ZM197M.PB7	0.11	0.073	1.8	0.022	0.11	0.073	0.023	0.15	0.094	0.023
ZM214M.PL15	0.12	0.17	0.069	0.10	0.052	0.062	0.15	0.063	0.080	0.057
ZM233M.PB6	0.27	0.26	3.0	0.085	0.36	0.41	0.076	0.30	0.30	0.089
ZM249M.PL1	0.19	0.16	0.15	0.100	0.094	0.070	0.58	0.51	0.66	0.19
CAP8.6F	0.54	5.1	0.056	0.51	0.042	0.053	0.45	0.041	0.052	0.040
CAP45.2.00.G3	0.34	6.2	35	0.14	0.34	7.5	0.14	0.33	4.5	0.14
CAP61.4.22.F10A	1.0	3.2	0.10	0.85	0.068	0.095	0.98	0.069	0.096	0.067
CAP63.A9	0.69	0.51	0.35	0.26	0.23	0.14	0.47	0.34	0.21	0.15
CAP84.32	0.98	17	0.012	0.78	0.012	0.012	0.80	0.012	0.012	0.012
CAP85.9	0.45	29	0.17	0.47	0.078	0.18	0.48	0.078	0.18	0.081
CAP88.B5	0.040	11	5.3	0.041	0.037	17	0.041	0.037	6.2	0.038
CAP206.8	0.32	2.1	>50	0.12	0.31	1.7	0.12	0.31	1.7	0.12
CAP210.2.00.E8	0.53	0.0097	46	0.0091	0.53	0.0097	0.013	2.0	0.015	0.013
CAP228.51	>50	47	>50	14	>50	47	14	>50	47	14
CAP239.2.00.G3J	0.49	1.0	0.17	0.12	0.060	0.055	0.13	0.060	0.055	0.032
CAP244.D3	0.68	4.4	4.3	0.74	0.72	10	1.0	1.0	16	1.2
CAP255.2.00.16J	0.73	5.1	0.026	0.78	0.025	0.027	1.8	0.026	0.029	0.027
CAP256.SU	0.29	0.012	0.047	0.013	0.050	0.0078	0.013	0.049	0.0079	0.0080
ConC	0.33	0.039	0.042	0.035	0.040	0.013	0.032	0.037	0.013	0.013
**Geometric mean**	**0.23**	**0.61**	**0.25**	**0.15**	**0.093**	**0.18**	**0.28**	**0.16**	**0.34**	**0.11**
**Breadth**	**98%**	**98%**	**93%**	**100%**	**98%**	**100%**	**100%**	**98%**	**93%**	**100%**

Supplementary Figure 5. Active coverage by scFv of a 45 virus panel

IC_{80} of scFv

	active coverage ≥ 1 scFv				active coverage ≥ 2 scFv		
	1μg/mL	10μg/mL	50μg/mL		1μg/mL	10μg/mL	50μg/mL
CAP256.25 +8ANC195	20%	58%	80%		4%	9%	20%
3BNC117 +8ANC195	40%	67%	80%		2%	22%	36%
PGT121 +8ANC195	47%	67%	82%		4%	18%	27%
CAP256.25 +3BNC117	51%	71%	89%		0%	16%	33%
CAP256.25 +PGT121	58%	69%	82%		7%	18%	33%
10E8v4+ 8ANC195	64%	98%	98%		0%	24%	40%
3BNC117 +PGT121	67%	82%	91%		13%	33%	40%
CAP256.25 +10E8v4	73%	98%	100%		9%	29%	49%
PGT121 +10E8v4	80%	98%	98%		20%	51%	56%
3BNC117 +10E8v4	93%	98%	98%		9%	62%	67%

	active coverage ≥ 1 scFv				active coverage ≥ 2 scFv		
	1μg/mL	10μg/mL	50μg/mL		1μg/mL	10μg/mL	50μg/mL
CAP256.25 +3BNC117 +8ANC195	53%	82%	98%		7%	33%	49%
CAP256.25 +PGT121 +8ANC195	58%	80%	93%		11%	27%	44%
3BNC117 +PGT121 +8ANC195	69%	84%	93%		16%	42%	53%
CAP256.25 +3BNC117 +PGT121	76%	89%	100%		20%	44%	53%
CAP256.25 +10E8v4 +8ANC195	82%	98%	100%		13%	44%	69%
PGT121 +10E8v4 +8ANC195	84%	98%	98%		24%	58%	69%
CAP256.25 +PGT121 +10E8v4	89%	98%	100%		27%	62%	71%
3BNC117+ 10E8v4+ 8ANC195	93%	98%	98%		11%	64%	71%
CAP256.25 +3BNC117 +10E8v4	96%	98%	100%		18%	76%	82%
3BNC117 +PGT121 +10E8v4	96%	98%	98%		38%	80%	82%

	active coverage ≥ 1 scFv				active coverage ≥ 2 scFv		
	1μg/mL	10μg/mL	50μg/mL		1μg/mL	10μg/mL	50μg/mL
CAP256.25 +3BNC117 +PGT121 +10E8v4	98%	98%	100%		44%	87%	91%
3BNC117 +PGT121 +10E8v4 +8ANC195	96%	98%	98%		40%	80%	84%
CAP256.25 +3BNC117 +10E8v4 +8ANC195	96%	98%	100%		24%	76%	87%
CAP256.25 +PGT121 +10E8v4 +8ANC195	91%	98%	100%		31%	69%	82%
CAP256.25 +3BNC117 +PGT121 +8ANC195	78%	93%	100%		22%	51%	64%

Supplementary Figure 6

A

Combination IC_{80} titres in µg/mL (Bliss-Hill model)

1 IgG

Virus name	CAP256.25	PGT121	3BNC117	10E8v4	8ANC195
Q23.17	0.098	0.080	0.062	8.133	50.000
Q168.A2	0.022	124.840	0.211	4.866	2.406
Q259.D2.17	1.285	27.253	0.096	21.218	>200.0
Q461.E2	0.723	>200.0	0.153	8.041	4.338
Q769.D22	>200.0	>200.0	0.053	3.698	1.121
Q842.D12	1.696	0.363	0.024	12.641	>200.0
BG505.W6M 332N	0.008	0.119	0.081	2.086	0.579
CAAN5342.A2	>200.0	0.156	2.319	13.333	>200.0
AC10.0.29	0.455	0.161	50.000	1.613	6.174
RHPA4259.7	>200.0	0.183	0.067	4.458	1.091
TRO.11	>200.0	0.139	0.104	0.723	1.030
PVO.4	0.470	1.290	0.213	24.136	1.070
SC422661.8	>200.0	0.730	0.167	2.008	2.530
REJO.67	>200.0	48.447	0.118	1.920	1.679
TRJO4551.18	>200.0	51.140	0.337	10.265	3.722
WITO.33	>200.0	9.087	0.104	1.124	>200.0
THRO4156.18	200.000	>200.0	7.715	1.905	>200.0
QH0692.42	>200.0	3.973	0.768	3.417	21.396
6535.3	>200.0	0.024	2.872	1.574	0.738
Du151.2	0.014	0.077	>200.0	1.674	>200.0
Du156.12	0.015	0.125	0.181	0.135	2.853
Du172.17	>200.0	0.794	3.573	0.648	50.000
Du422.1	0.482	0.429	>200.0	1.740	>200.0
ZM53M.PB12	0.004	0.006	0.841	11.917	64.414
ZM109F.PB4	0.592	31.281	0.499	2.272	>200.0
ZM135M.PL10A	>200.0	8.443	0.216	1.101	>200.0
ZM197M.PB7	0.026	>200.0	1.717	0.495	179.270
ZM214M.PL15	1.913	0.476	0.886	5.674	2.843
ZM233M.PB6	0.002	14.296	0.913	1.116	28.922
ZM249M.PL1	1.942	5.816	0.573	4.316	3.551
CAP8.6F	24.300	0.042	200.000	6.754	15.793
CAP45.2.00.G3	0.011	50.000	14.103	3.743	200.000
CAP61.4.22.F10A	6.664	0.151	0.347	12.178	28.991
CAP63.A9	0.090	0.411	0.984	2.522	>200.0
CAP84.32	22.521	0.022	0.286	4.706	>200.0
CAP85.9	>200.0	0.359	5.292	2.818	200.000
CAP88.B5	>200.0	2.773	5.562	0.135	27.049
CAP206.8	0.094	9.859	>200.0	0.973	>200.0
CAP210.2.00.E8	0.007	93.630	50.000	3.907	>200.0
CAP228.51	24.176	71.030	0.161	104.456	>200.0
CAP239.2.00.G3J	0.011	0.022	>200.0	5.753	>200.0
CAP244.D3	>200.0	>200.0	0.500	2.411	12.772
CAP255.2.00.16J	>200.0	0.101	0.096	3.900	64.601
CAP256.SU	0.006	0.126	0.562	7.148	>200.0
ConC	0.004	0.045	0.144	4.127	50.000
**Geometric mean**	**0.19**	**0.89**	**0.59**	**3.19**	**9.08**
**Breadth**	**64%**	**89%**	**91%**	**100%**	**64%**

2 IgG Combinations

Virus name	3BNC117 +8ANC195	3BNC117 +10E8v4	CAP256.25 +3BNC117	PGT121 +3BNC117	10E8v4 +8ANC195	CAP256.25 +8ANC195	PGT121 +8ANC195	CAP256.25 +10E8v4	PGT121 +10E8v4	CAP256.25 +PGT121
Q23.17	0.061	0.062	0.035	0.039	4.044	0.077	0.072	0.091	0.078	0.008
Q168.A2	0.171	0.172	0.016	0.210	0.945	0.020	2.280	0.019	4.259	0.022
Q259.D2.17	0.096	0.095	0.041	0.096	21.218	1.285	27.253	0.844	11.189	1.213
Q461.E2	0.129	0.143	0.049	0.153	1.741	0.284	4.338	0.419	8.041	0.723
Q769.D22	0.041	0.051	0.053	0.053	0.592	1.121	1.121	3.698	3.698	>200.0
Q842.D12	0.024	0.024	0.011	0.019	12.641	1.696	0.363	0.921	0.341	0.079
BG505.W6M 332N	0.053	0.065	0.006	0.040	0.260	0.007	0.074	0.007	0.094	0.007
CAAN5342.A2	2.319	1.622	2.319	0.143	13.333	>200.0	0.156	13.333	0.149	0.156
AC10.0.29	4.559	1.523	0.443	0.161	0.680	0.189	0.135	0.155	0.129	0.064
RHPA4259.7	0.057	0.057	0.067	0.037	0.480	1.091	0.119	4.458	0.125	0.183
TRO.11	0.071	0.066	0.104	0.054	0.232	1.030	0.104	0.723	0.097	0.139
PVO.4	0.126	0.192	0.074	0.129	0.726	0.132	0.287	0.331	0.825	0.137
SC422661.8	0.116	0.134	0.167	0.128	0.543	2.530	0.358	2.008	0.407	0.730
REJO.67	0.085	0.106	0.118	0.088	0.545	1.679	0.623	1.920	0.928	48.447
TRJO4551.18	0.253	0.311	0.337	0.315	1.889	3.722	2.450	10.265	5.475	51.140
WITO.33	0.104	0.083	0.104	0.078	1.124	>200.0	9.087	1.124	0.524	9.087
THRO4156.18	7.715	1.125	4.941	7.715	1.905	200.000	>200.0	1.466	1.905	200.000
QH0692.42	0.649	0.375	0.768	0.382	1.843	21.396	1.998	3.417	0.726	3.973
6535.3	0.380	0.498	2.872	0.023	0.212	0.738	0.017	1.574	0.016	0.024
Du151.2	>200.0	1.674	0.014	0.077	1.674	0.014	0.077	0.013	0.058	0.006
Du156.12	0.138	0.051	0.013	0.045	0.103	0.015	0.093	0.010	0.033	0.009
Du172.17	3.049	0.310	3.573	0.289	0.632	50.000	0.760	0.648	0.113	0.794
Du422.1	>200.0	1.740	0.482	0.429	1.740	0.482	0.429	0.167	0.207	0.039
ZM53M.PB12	0.835	0.739	0.004	0.006	9.110	0.004	0.006	0.004	0.006	0.002
ZM109F.PB4	0.499	0.169	0.075	0.492	2.272	0.592	31.281	0.058	1.886	0.560
ZM135M.PL10A	0.216	0.098	0.216	0.192	1.101	>200.0	8.443	1.101	0.625	8.443
ZM197M.PB7	1.495	0.224	0.025	1.717	0.451	0.026	179.270	0.016	0.495	0.026
ZM214M.PL15	0.506	0.310	0.081	0.119	1.065	0.455	0.338	0.182	0.200	0.051
ZM233M.PB6	0.901	0.295	0.002	0.710	1.079	0.002	7.911	0.002	0.714	0.002
ZM249M.PL1	0.573	0.221	0.071	0.307	2.481	1.747	2.706	0.140	0.819	0.269
CAP8.6F	9.171	4.505	6.839	0.042	2.125	1.924	0.041	0.856	0.038	0.028
CAP45.2.00.G3	5.335	1.025	0.010	1.837	1.881	0.010	5.860	0.008	0.638	0.006
CAP61.4.22.F10A	0.347	0.292	0.119	0.084	11.949	6.655	0.151	0.989	0.139	0.066
CAP63.A9	0.984	0.435	0.060	0.209	2.522	0.090	0.411	0.066	0.253	0.049
CAP84.32	0.286	0.220	0.150	0.020	4.706	22.521	0.022	1.085	0.022	0.018
CAP85.9	3.611	1.136	5.292	0.321	1.775	200.000	0.326	2.818	0.217	0.359
CAP88.B5	2.101	0.095	5.562	0.754	0.115	27.049	1.320	0.135	0.080	2.773
CAP206.8	>200.0	0.973	0.094	9.859	0.973	0.094	9.859	0.051	0.853	0.093
CAP210.2.00.E8	50.000	3.021	0.007	22.462	3.907	0.007	93.630	0.007	3.460	0.007
CAP228.51	0.161	0.161	0.057	0.161	104.456	24.176	71.030	13.234	42.192	7.583
CAP239.2.00.G3J	>200.0	5.753	0.011	0.022	5.753	0.011	0.022	0.010	0.020	0.006
CAP244.D3	0.399	0.219	0.500	0.500	1.084	12.772	12.772	2.411	2.411	>200.0
CAP255.2.00.16J	0.092	0.081	0.096	0.040	2.314	64.601	0.098	3.900	0.088	0.101
CAP256.SU	0.562	0.540	0.006	0.126	7.148	0.006	0.126	0.006	0.105	0.004
ConC	0.143	0.138	0.004	0.032	3.334	0.004	0.045	0.004	0.044	0.004
**Geometric mean**	**0.42**	**0.28**	**0.10**	**0.16**	**1.66**	**0.48**	**0.68**	**0.24**	**0.33**	**0.13**
**Breadth**	**91%**	**100%**	**100%**	**100%**	**100%**	**93%**	**98%**	**100%**	**100%**	**96%**

3 IgG Combinations

Virus name	3BNC117 +10E8v4 +8ANC195	CAP256.25 +3BNC117 +8ANC195	PGT121 +3BNC117 +8ANC195	CAP256.25 +3BNC117 +10E8v4	PGT121 +3BNC117 +10E8v4	CAP256.25 +PGT121 +3BNC117	CAP256.25 +10E8v4 +8ANC195	PGT121 +10E8v4 +8ANC195	CAP256.25 +PGT121 +8ANC195	CAP256.25 +PGT121 +10E8v4
Q23.17	0.061	0.034	0.038	0.035	0.038	0.008	0.073	0.070	0.008	0.008
Q168.A2	0.146	0.015	0.171	0.015	0.171	0.016	0.018	0.930	0.020	0.019
Q259.D2.17	0.095	0.041	0.096	0.041	0.095	0.041	0.844	11.189	1.213	0.828
Q461.E2	0.122	0.046	0.129	0.048	0.143	0.049	0.234	1.741	0.284	0.419
Q769.D22	0.040	0.041	0.041	0.051	0.051	0.053	0.592	0.592	1.121	3.698
Q842.D12	0.024	0.011	0.019	0.011	0.019	0.010	0.921	0.341	0.079	0.078
BG505.W6M 332N	0.046	0.006	0.032	0.006	0.036	0.006	0.007	0.063	0.006	0.007
CAAN5342.A2	1.622	2.319	0.143	1.622	0.138	0.143	13.333	0.149	0.156	0.149
AC10.0.29	0.669	0.188	0.135	0.155	0.128	0.064	0.108	0.112	0.056	0.055
RHPA4259.7	0.050	0.057	0.034	0.057	0.033	0.037	0.480	0.092	0.119	0.125
TRO.11	0.052	0.071	0.046	0.066	0.044	0.054	0.232	0.078	0.104	0.097
PVO.4	0.119	0.055	0.090	0.070	0.121	0.055	0.119	0.250	0.078	0.123
SC422661.8	0.099	0.116	0.097	0.134	0.110	0.128	0.543	0.246	0.358	0.407
REJO.67	0.079	0.085	0.066	0.106	0.081	0.088	0.545	0.330	0.623	0.928
TRJO4551.18	0.240	0.253	0.242	0.311	0.293	0.315	1.889	1.524	2.450	5.475
WITO.33	0.083	0.104	0.078	0.083	0.066	0.078	1.124	0.524	9.087	0.524
THRO4156.18	1.125	4.941	7.715	0.961	1.125	4.941	1.466	1.905	>200	1.466
QH0692.42	0.346	0.649	0.351	0.375	0.237	0.382	1.843	0.608	1.998	0.726
6535.3	0.163	0.380	0.017	0.498	0.015	0.023	0.212	0.013	0.017	0.016
Du151.2	1.674	0.014	0.077	0.013	0.058	0.006	0.013	0.058	0.006	0.006
Du156.12	0.047	0.012	0.041	0.009	0.023	0.008	0.010	0.030	0.009	0.007
Du172.17	0.308	3.049	0.287	0.310	0.088	0.289	0.632	0.112	0.760	0.113
Du422.1	1.740	0.482	0.429	0.167	0.207	0.039	0.167	0.207	0.039	0.034
ZM53M.PB12	0.736	0.004	0.006	0.004	0.006	0.002	0.004	0.006	0.002	0.002
ZM109F.PB4	0.169	0.075	0.492	0.032	0.169	0.075	0.058	1.886	0.560	0.058
ZM135M.PL10A	0.098	0.216	0.192	0.098	0.093	0.192	1.101	0.625	8.443	0.625
ZM197M.PB7	0.216	0.024	1.495	0.016	0.224	0.025	0.016	0.451	0.026	0.016
ZM214M.PL15	0.257	0.077	0.112	0.049	0.080	0.026	0.153	0.178	0.050	0.034
ZM233M.PB6	0.294	0.002	0.704	0.002	0.265	0.002	0.002	0.704	0.002	0.002
ZM249M.PL1	0.221	0.071	0.307	0.041	0.157	0.055	0.140	0.819	0.269	0.086
CAP8.6F	1.832	1.572	0.041	0.758	0.038	0.028	0.538	0.037	0.027	0.026
CAP45.2.00.G3	0.747	0.009	1.126	0.007	0.336	0.006	0.007	0.461	0.006	0.005
CAP61.4.22.F10A	0.292	0.119	0.084	0.109	0.081	0.045	0.989	0.139	0.066	0.063
CAP63.A9	0.435	0.060	0.209	0.050	0.160	0.040	0.066	0.253	0.049	0.042
CAP84.32	0.220	0.150	0.020	0.127	0.019	0.016	1.085	0.022	0.018	0.018
CAP85.9	0.927	3.611	0.294	1.136	0.204	0.321	1.775	0.202	0.326	0.217
CAP88.B5	0.085	2.101	0.549	0.095	0.064	0.754	0.115	0.073	1.320	0.080
CAP206.8	0.973	0.094	9.859	0.051	0.853	0.093	0.051	0.853	0.093	0.051
CAP210.2.00.E8	3.021	0.007	22.462	0.007	2.784	0.007	0.007	3.460	0.007	0.007
CAP228.51	0.161	0.057	0.161	0.057	0.161	0.057	13.234	42.192	7.583	7.018
CAP239.2.00.G3J	5.753	0.011	0.022	0.010	0.020	0.006	0.010	0.020	0.006	0.006
CAP244.D3	0.196	0.399	0.399	0.219	0.219	0.500	1.084	1.084	12.772	2.411
CAP255.2.00.16J	0.078	0.092	0.040	0.081	0.038	0.040	2.314	0.086	0.098	0.088
CAP256.SU	0.540	0.006	0.126	0.006	0.105	0.004	0.006	0.105	0.004	0.004
ConC	0.137	0.004	0.032	0.004	0.031	0.004	0.004	0.044	0.004	0.004
**Geometric mean**	**0.24**	**0.08**	**0.15**	**0.06**	**0.10**	**0.04**	**0.16**	**0.25**	**0.10**	**0.07**
**Breadth**	**100%**	**100%**	**100%**	**100%**	**100%**	**100%**	**100%**	**100%**	**98%**	**100%**

B

Active coverage by IgG of a 45 virus panel

		IgG IC_{80}, Bliss Hill model									
		3BNC117 +10E8v4	PGT121 +10E8v4	CAP256.25 +10E8v4	10E8v4 +8ANC195	PGT121 +3BNC117	CAP256.25 +3BNC117	CAP256.25 +PGT121	PGT121 +8ANC195	CAP256.25 +8ANC195	3BNC117 +8ANC195
active coverage ≥ 1 scFv	50µg/mL	100%	100%	100%	100%	100%	100%	96%	98%	93%	91%
	10µg/mL	100%	96%	93%	89%	98%	100%	89%	84%	73%	89%
	1µg/mL	78%	78%	64%	33%	89%	84%	76%	58%	49%	69%
active coverage ≥ 2 scFv	50µg/mL	82%	78%	60%	47%	64%	47%	49%	36%	24%	42%
	10µg/mL	64%	58%	44%	29%	56%	40%	38%	22%	18%	31%
	1µg/mL	4%	7%	7%	0%	33%	27%	24%	4%	2%	2%

		PGT121 +3BNC117 +10E8v4	CAP256.25 +3BNC117 +10E8v4	3BNC117 +10E8v4 +8ANC195	PGT121 +10E8v4 +8ANC195	CAP256.25 +10E8v4 +8ANC195	CAP256.25 +PGT121 +10E8v4	PGT121 +3BNC117 +8ANC195	CAP256.25 +PGT121 +3BNC117	CAP256.25 +3BNC117 +8ANC195	CAP256.25 +PGT121 +8ANC195
active coverage ≥ 1 scFv	50µg/mL	100%	100%	100%	100%	100%	100%	100%	100%	100%	98%
	10µg/mL	100%	100%	100%	96%	96%	100%	98%	100%	100%	96%
	1µg/mL	96%	96%	84%	82%	76%	89%	89%	98%	87%	78%
active coverage ≥ 2 scFv	50µg/mL	96%	100%	87%	89%	82%	89%	80%	89%	73%	69%
	10µg/mL	89%	91%	71%	69%	60%	82%	69%	80%	58%	51%
	1µg/mL	36%	33%	7%	11%	9%	33%	36%	53%	27%	27%
